# DNA methylome analysis reveals novel insights into active hypomethylated regulatory mechanisms of temperature-dependent flower opening in *Osmanthus fragrans*

**DOI:** 10.1093/hr/uhae010

**Published:** 2024-01-10

**Authors:** Shiwei Zhong, Huijun Zhu, Wenle Li, Dan Wu, Yunfeng Miao, Bin Dong, Yiguang Wang, Zhen Xiao, Qiu Fang, Jinping Deng, Hongbo Zhao

**Affiliations:** Zhejiang Provincial Key Laboratory of Germplasm Innovation and Utilization for Garden Plants, Key Laboratory of National Forestry and Grassland Administration on Germplasm Innovation and Utilization for Southern Garden Plants, School of Landscape and Architecture, Zhejiang A&F University, Hangzhou, Zhejiang 311300, China; Zhejiang Provincial Key Laboratory of Germplasm Innovation and Utilization for Garden Plants, Key Laboratory of National Forestry and Grassland Administration on Germplasm Innovation and Utilization for Southern Garden Plants, School of Landscape and Architecture, Zhejiang A&F University, Hangzhou, Zhejiang 311300, China

## Abstract

Short-term ambient low temperature (ALT) stimulation is necessary for *Osmanthus fragrans* to facilitate continued flower opening after floral bud development reaches maturity. DNA methylation, a vital epigenetic modification, regulates various biological processes in response to temperature fluctuations. However, its role in temperature-driven flower opening remains elusive. In this study, we identified the pivotal timeframe during which *O. fragrans* promptly detected temperature cues. Using whole-genome bisulfite sequencing, we explored global DNA hypomethylation during this phase, with the most significant changes occurring in CHH sequence contexts. Auxin transport inhibitor (TIBA) application revealed that ALT-induced endogenous auxin accumulation promoted peduncle elongation. In our mRNA-seq analysis, we discovered that the differentially expressed genes (DEGs) with hypo-differentially methylated regions (hypo-DMRs) were mainly enriched in auxin and temperature response, RNA processing, and carbohydrate and lipid metabolism. Transcripts of three DNA demethylase genes (*OfROS1a*, *OfDML3*, *OfDME*) showed upregulation. Furthermore, all DNA methylase genes, except *OfCMT2b*, also displayed increased expression, specifically with two of them, *OfCMT3a* and *OfCMT1*, being associated with hypo-DMRs. Promoter assays showed that *OfROS1a*, with promoters containing low-temperature- and auxin-responsive elements, were activated by ALT and exogenous IAA at low concentrations but inhibited at high concentrations. Overexpression of *OfROS1* reduced endogenous auxin levels but enhanced the expression of genes related to auxin response and spliceosome in *petunia*. Furthermore, *OfROS1* promoted sucrose synthesis in petunia corollas. Our data characterized the rapid response of active DNA hypomethylation to ALT and suggested a possible epiregulation of temperature-dependent flower opening in *O. fragrans*. This study revealed the pivotal role of DNA hypomethylation in *O. fragrans* during the ALT-responsive phase before flower opening, involving dynamic DNA demethylation, auxin signaling modulation, and a potential feedback loop between hypomethylation and methylation.

## Introduction

Flowering, typically divided into initiation, floral bud differentiation, flower opening, and senescence stages, is a pivotal phase in the reproductive cycle of flowering plants, enabling pollination and fertilization by facilitating access to reproductive organs for both pollinators and wind dispersion [1, 2]. This intricate process involves a series of physiological and biochemical changes driven by internal and external factors, such as temperature, light, hormones, and genetic components [3–5]. Notably, temperature plays a central role in various aspects of angiosperm flowering, including temperature-sensitive flower initiation, vernalization, bud dormancy release, and flowering rate regulation [6–9]. Flower opening, indicated by visible anthers and pistils, driven by coordinated floral organ development, especially petals, is crucial for both aesthetics and economic value [10]. The process of flower opening involves a sequence of coordinated events, beginning with cell enlargement in the petals, which leads to the subsequent expansion of the petal area. This growth, unevenly distributed between the abaxial and adaxial sides of the petal base, ultimately results in the coordinated movement of the petals, facilitating the seamless transition from a closed bud to a fully opened flower [10].

Temperature exerts its influence on plant growth and development through various mechanisms, including transcriptional regulation and epigenetic modifications [11, 12]. Among these, DNA methylation, a critical epigenetic alteration, has been recognized for its involvement in a wide array of temperature-dependent biological processes [13]. For instance, storage at the specific temperature of 16°C led to reduced methylation levels in the promoter region of genes associated with anthocyanin accumulation in peach [14]. Moreover, DNA demethylation regulation significantly enhanced cold tolerance, enabling the successful expansion of *Hevea brasiliensis* to high-latitude regions [15]. Additionally, DNA methylation also governs various temperature-dependent stages of the flowering process. The increased petal number in *Rosa hybrida* induced by low temperatures, along with the chilling requirements and dormancy break in floral buds of many woody perennials, were all controlled by DNA hypermethylation or hypomethylation regulation [16–18]. However, there are no reports describing the involvement of DNA methylation in the rapid response of flower opening to ambient low temperature (ALT) in plants, in contrast to dormant floral buds, which are slower in perceiving temperature signals.

DNA methylation occurs at the 5′ position of cytosine in various cytosine sequence contexts (CG, CHG, and CHH), contributing to the regulation of nuclear gene expression and genome stability [19]. DNA methyltransferases, including methyltransferase 1 (MET1), chromomethylases (CMT2 and CMT3), and domains rearranged methylases (DRM1 and DRM2), maintain global DNA methylation in plants [20–24]. Enzymatic erasure of DNA methylation, aside from passive demethylation due to DNA methyltransferase deficiency or lack of methyl donors, is facilitated by a family of four bifunctional 5-methylcytosine (5-mC) DNA glycosylases: repressor of silencing 1 (ROS1), transcriptional activator Demeter (DME), and Demeter-like proteins (DML2 and DML3) [25]. DNA methylases and demethylases involved in regulating DNA methylation for a specific biological process might exhibit diversity or potential cooperation. For instance, DNA hypomethylation resulting from both the *MET1* deficiency of *Capsicum annuum* and *ROS1* activation of *Malus domestica* promoted fruit ripening [26, 27]. Additionally, *DML2* notably accelerated tomato fruit ripening as well [28]. Furthermore, a DNA methylase or demethylase was not restricted to a distinct biological process but played a crucial role in multiple life events. In *Arabidopsis thaliana*, *Atmet1* mutations also led to developmental anomalies [29], and *Atros1* was more susceptible to cold stress and negatively regulates seed dormancy [30, 31]. *DML2* has not only been reported as essential for maintaining the proper distribution of 5-methylcytosine (5-mC) in methylated sequences [32], but also for its involvement in the regulation of DNA hypomethylation during flower opening [33].

DNA methylation dynamics, modulating chromatin structure and transcriptional activity, commonly regulates various developmental stages by mediating auxin signals, given the essential role of the phytohormone auxin in plant development from embryogenesis to senescence [34–36]. Auxin application caused significant transcriptional differences in genes encoding DNA methylases or demethylases in *A. thaliana*, such as direct modulation of *AtCMT3* transcription through the presence of an auxin-responsive element (AuxRE motif) in its promoter and indirect effects on *AtCMT1*[37–39]. Exogenous IAA application to mature-green pericarp discs inhibited fruit ripening mediated by the upregulated expression of *CaMET1* and *CaCMT3* in *C. annuum* [26]. Conversely, DNA methylation also had an impact on auxin signaling components, as evidenced by studies showing that *Atmet1* and *Atmet1-Atcmt3* embryos yield abnormal auxin gradients [40], and *CaMET1* suppression in *C. annuum* resulted in inhibited auxin signaling and decreased auxin levels [26].

*Osmanthus fragrans*, a perennial woody plant native to China, is renowned for its distinctive fragrance, making it a valuable asset in landscaping, natural flavor enhancement, and perfume oil production [41, 42]. However, the variable temperatures across regions pose a challenge to achieving synchronized flower opening in *O. fragrans*, impacting its ornamental and economic value. In previous research, we identified the significance of a specific low-temperature phase following floral bud formation for successful flower opening in *O. fragrans* [43]. Yet the molecular basis of temperature-responsive flower opening remained elusive. In this study, we utilized whole-genome bisulfite sequencing libraries to capture the entire cytosine methylation landscape. We integrated methylome and transcriptome analyses on floral buds treated at 19°C for different durations in *O. fragrans*. Exposure to 19°C led to notable reductions in DNA methylation levels, implying a regulatory role for hypomethylation in temperature-dependent flower opening. We scrutinized the impact of ALT on hypo-differentially methylated regions (hypo-DMRs) linked to differentially expressed genes (DEGs) involved in auxin signaling, lipid and carbohydrate metabolism, and related processes. Furthermore, we elucidated the biological function of *OfROS1a* and deeply investigated the DNA demethylation-driven temperature response mechanism preceding flower opening.

## Results

### Assessment of temperature-dependent flower opening in *O. fragrans*

**Figure 1:**
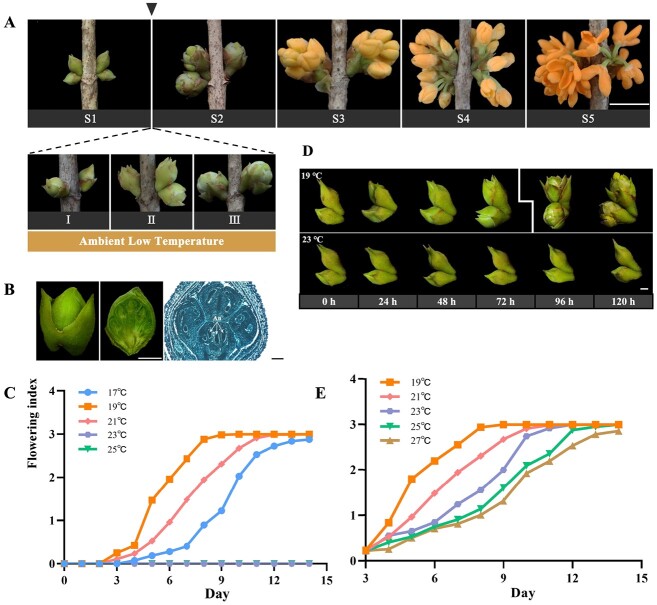
Phenotypes of temperature-dependent flower opening in *O. fragrans.***A** Five stages of flower opening in sweet osmanthus: S1, the outer bud scales have unfurled, and the inner bud scales are still furled; S2, the bud has become globular-shaped and the inside bracts covering the inflorescence are visible; S3, the inflorescence has burst through the bracts and the florets are closely crowded; S4, initial flowering stage; S5, full flowering stage. Scale bar = 1 cm. **B** Phenotype (left), dissection (middle), and oblique structural section (right) of mature floral buds at stage 1. Scale bar = 1 mm (left and middle), 1 μm (right). An, anther. **C** Rate analysis of the flower opening process under 17, 19, 21, 23, or 25°C treatments after floral bud development reached maturity in *O. fragrans*. **D** Phenotypes of floral buds treated at 19°C (top) and 23°C (bottom) for 24, 48, 72, 96, or 120 h. Scale bar = 1 mm. **E** Rate analysis of the flower opening process under 19, 21, 23, 25, or 27°C conditions post 72-hour 19°C treatment, showing outer bud bract splitting.

To give a precise description of the process of flower opening in *O. fragrans*, we characterized different opening stages and the effects of environmental temperature on opening. As illustrated in Fig. 1A, flower opening in *O. fragrans* comprises five sequential stages: stage 1 (S1) involves the unfolding of outer bud scales while the inner scales remain closed; stage 2 (S2) is marked by the transition to a globular bud shape and the emergence of inner bracts covering the inflorescence; stage 3 (S3) corresponds to the breakthrough of the inflorescence, leading to densely clustered florets; stage 4 (S4) signifies the initial phase of flowering; and stage 5 (S5) represents full flowering. Our previous investigation showed that the transition from floral bud development reaching maturity, characterized by stamen differentiation and pistil degeneration in Fig. 1B, to full blooming in S5, was induced by ALT [43].

For our study, we utilized a male *O. fragrans* specimen of the cultivar ‘Yanhong Gui’ to assess the impact of temperature on flower opening. Notably, the fastest flower opening occurred at a temperature of 19°C in *O. fragrans* (Fig. 1C). Interestingly, the time taken for *O. fragrans* to progress from floral buds (S1) to full blooming (S5) was extended under both higher (22, 21, 20°C) and lower (18, 17°C) temperature treatments (Fig. 1C). This indicates that while temperature influenced flower opening, the flowering rate did not rise with decreasing ambient temperature, suggesting a favorable temperature is required for *O. fragrans*. Furthermore, exposing floral buds to a controlled environment at 23°C resulted in a persistent inability to open, highlighting an ALT-responsive mechanism that triggers flower opening after floral bud development reaches maturity in *O. fragrans* (Fig. 1C). We conducted sequential observations of mature floral buds treated at 23 and 19°C, at a 24-h interval between treatments (Fig. 1D). Notably, a visible splitting of the outermost bud bracts covering the inflorescence was observed after 72 h of the 19°C treatment, as depicted on the left side of Fig. 1D. This event marked a continuous progression of the flower opening process, even at temperatures exceeding 23°C, indicating that the timeframe of 0 to 72 h during 19°C treatment was a crucial window period governing temperature-dependent floral bud opening (Fig. 1D and E).

### Features of genome-wide methylation of floral buds in *O. fragrans*

To determine the characteristic features and patterns of DNA methylation within different floral bud processing times at 19°C in sweet osmanthus cultivar ‘Yanhong Gui’, single-base resolution maps of DNA methylation via whole-genome bisulfite sequencing was performed for floral buds treated at 19°C for 24 h (L24), 48 h (L48), or 72 h (L72) and treated at 23°C (H) as controls, facilitating a thorough examination of the most substantial genome-wide alterations in DNA methylation. Each sample generated around 96–111 million raw reads, producing data exceeding 30 Gb. Approximately 71% of the reads were successfully aligned to the reference genome of sweet osmanthus (2*n* = 46) [44]. To ensure precision in DNA methylation analysis, we conducted three biological replicates for each sample, achieving an average coverage of over 15-fold and a bisulfite seqencing conversion rate of 99.5% (Supplementary Data Table S1). In *O. fragrans*, the chromosomal distribution of DNA methylation, reflecting the density of methylated cytosines (mCs), was consistent among all floral buds subjected to different treatments. Notably, the lowest proportion of methylation was observed in methylated CHG (18.7–21.0%) contexts, while CHH methylation had the highest occurrence (47.6–53.6%) (Supplementary Data Table S2, Supplementary Data Fig. S1). In contrast, mean methylation levels in the CG contexts were the highest, ranging from 57.9 to 63.6%, followed by CHG contexts (27.6–32.3%) (Supplementary Data Fig. S2). Supplementary Data Fig. S2A illustrates that methylation levels tended to be lower within regions of high gene density across the entire chromosomes. Furthermore, the distribution of DNA methylation levels varied among different gene features, including 5′ and 3′ flanking regions of genes, exons, introns, CpG islands (CGIs), regions 2 kb upstream and downstream of CGIs, known as CGI shores, and repeat regions (Fig. S2B). Across the three sequence contexts, relatively higher methylation levels were evident in repeat regions predominantly composed of transposable elements (TEs) [45], which was especially characterized by enrichment of mCG and mCHG from a global perspective (Supplementary Data Fig. S2).

**Figure 2:**
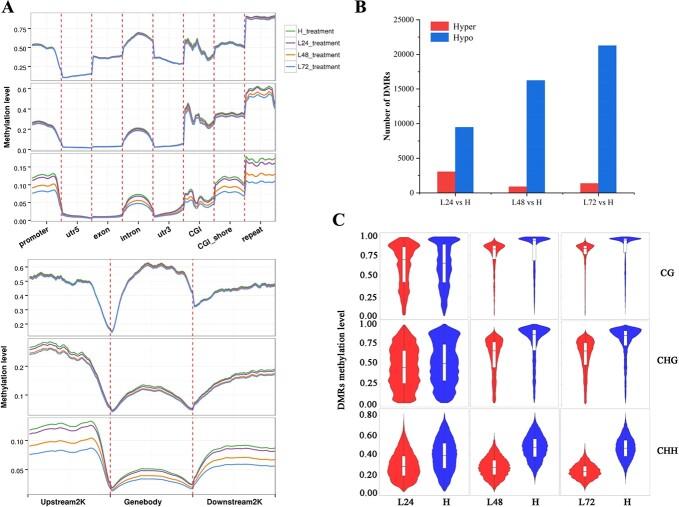
DNA methylation dynamics in floral buds of *O. fragrans* responding to ambient low temperature. **A** DNA methylation profiles of CG, CHG, and CHH among gene features including promoter, 5′ UTRs, exon, intron, 3′ UTRs, CGI, 2 kb upstream and downstream of CGI (CGI_shore), and repeat region (up), among gene bodies and their 2 kb upstream and downstream regions (bottom) in floral buds treated at 19°C for 24 (L24), 48 (L48), or 72 (L72) h relative to floral buds treated at 23°C (H) used as controls in *O. fragrans*. **B** Numbers of hyper- and hypo-DMRs in floral buds of L24, L48, and L72 relative to H. **C** Violin boxplots showing methylation levels of DMRs in the CG, CHG, and CHH contexts of different comparisons.

### Ambient low temperature induced DNA hypomethylation before flower opening

In our study, we observed distinct DNA demethylation patterns in response to ALT within 72 h in floral buds of the sweet osmanthus cultivar ‘Yanhong Gui’ (Supplementary Data Fig. S3; Fig. 2). For a deeper understanding of DNA methylation dynamics during temperature-dependent flower opening in *O. fragrans*, we conducted whole-genome methylation analysis on floral buds from H, L24, L48, and L72. Methylation levels in CG, CHG and CHH contexts overall trended highest in H, followed by L24 and L48, reaching their lowest in L72 (Supplementary Data Fig. S3). Interestingly, a comparative examination of DNA methylation levels across various genomic regions unveiled a consistent trend of lower methylation levels in floral buds as the treatment duration at 19°C increased (Fig. 2A). This reduction was most prominent in the CHG and CHH contexts within the 5′ flanking regions of gene bodies, and mainly occurred within the promoter regions and CGIs rather than the 5′ untranslated regions (UTRs) (Fig. 2A). No significant differences were observed in methylation patterns within exonic and 3′ UTR regions across floral buds subjected to different treatments (Fig. 2A). Despite the CHH context’s lowest methylation, its substantial reduction (5.2, 22.3, 34.1%) was key in lowering DNA methylation in L24, L48, and L72 compared with H, especially in CGI shores, defined as regions flanking CGIs with low CpG density, and repeat regions. This suggests that methylated cytosines in the CHH context might hold significance in activating flower opening in response to temperature fluctuations in *O. fragrans* (Supplementary Data Fig. S3, Fig. 2A).

Through a comparison of fractional methylation levels between L24 versus H, L48 versus H, and L72 versus H, we identified 3047, 894, and 1357 hyper-differentially methylated regions (hyper-DMRs), as well as 9462, 16 225, and 21 263 hypo-DMRs (Fig. 2B). Notably, as treatment duration at 19°C increased, the number of hypo-DMRs in L24, L48, and L72 floral buds relative to H steadily grew, while the number of hyper-DMRs in L48 and L72 relative to H remained lower than in L24 versus H (Fig. 2B). Across the three methylation contexts, both hypo- and hyper-DMRs were prominent in CGI shores, promoters, and introns (Supplementary Data Fig. S4). Additionally, differential DNA methylation at CG and CHG sites was enriched in CGI and exon regions (Supplementary Data Fig. S4). A comparative analysis revealed that DMRs in L24 floral buds exhibited a distributed range of DNA methylation levels (Fig. 2C). However, in L48 and L72 floral buds, CG-DMRs and CHG-DMRs were predominantly situated at relatively high methylation levels (Fig. 2C). DMRs in all three contexts displayed lower methylation levels in ALT-induced floral buds (19°C) than in those consistently maintained at 23°C, underlining temperature’s pivotal role in DNA methylation regulation for flower opening capacity in *O. fragrans* (Fig. 2C).

**Figure 3:**
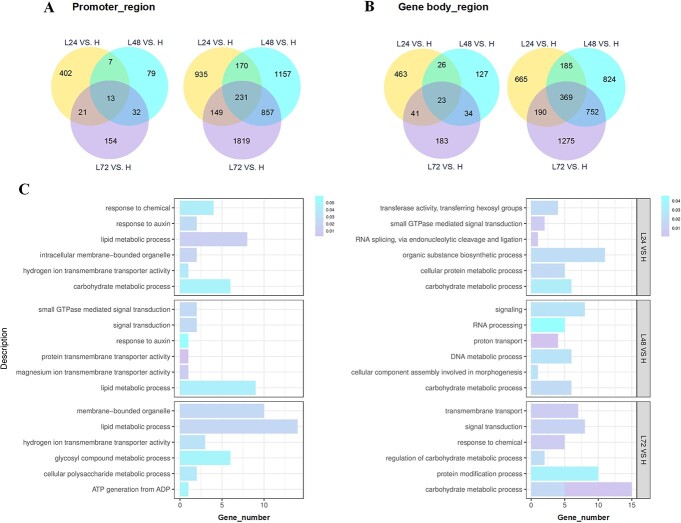
Analysis of DNA hypomethylation within 72 h at 19°C with temperature-dependent flower opening in *O. fragrans*. **A** Venn diagrams indicating the number of DMRs in promoter regions (DMR promoter genes) in floral buds for three different comparisons. **B** Venn diagrams indicating the number of DMRs in gene body regions (DMR genes) in floral buds for three different comparisons. **C** GO-based enrichment analysis of hypo-DMRs related to DEGs in the promoter region (left) and in the gene body region (right).

### Gene Ontology enrichment analysis of hypo-DMR-related genes in floral buds with diverse treatment duration in the ambient low temperature condition

Venn diagrams revealed that within various comparison groups (L24 versus H, L48 versus H, and L72 versus H), 1928, 2546, and 3276 genes were associated with DMRs in the promoters (referred to as DMR promoter genes), while 1962, 2340, and 2867 genes were associated with DMRs in the gene bodies (referred to as DMR genes) (Fig. 3A and B). These findings signified dynamic changes in fractional methylation levels within functional regions of floral buds activated for flower opening through 19°C inductions. Notably, in all combinations, hypo-DMR promoter genes (77.0, 94.9, 93.3%) and hypo-DMR genes (73.2, 91.0, 90.2%) emerged as predominant categories (Fig. 3A and B). Consequently, we conducted functional characterization of hypo-DMR promoter genes and hypo-DMR genes associated with DEGs using Gene Ontology (GO) enrichment analysis across floral buds subjected to 19°C treatment every 24 h in *O. fragrans* (Fig. 3C). Examination of enriched GO domain terms revealed substantial enrichment of hypo-DMR-associated DEGs, encompassing both hypo-DMR promoter and hypo-DMR DEG categories, in the biological processes related to lipid metabolism and carbohydrate metabolism across all comparisons (L24 versus H, L48 versus H, and L72 versus H), implying an important involvement in metabolite accumulation or consumption during the preparatory phase preceding flower opening (Fig. 3C). Notably, several critical GO terms associated with signal response and biological signal transduction, including response to auxin, response to chemical, signal transduction, and small GTPase-mediated signal transduction, were discerned within floral buds subjected to varying durations of 19°C treatment, implying that DNA hypomethylation regulation triggered by ALT could hold pivotal significance in perceiving and reacting to signals within plant systems (Fig. 3C). Furthermore, hypo-DMR-associated DEGs exhibited enrichment in processes related to protein modification and RNA processing, particularly evident in the context of L48 versus H and L72 versus H comparisons (Fig. 3C, Supplementary Data Tables S3 andS4). The analysis of molecular functions revealed that DEGs associated with transporter activities, including oxidoreductase activity, hydrogen ion transmembrane transporter activity, and magnesium ion transmembrane transporter activity, displayed enrichment within the hypo-DMR gene pool, which concurred with the effects of the 19°C treatments on biological processes involving proton and ion transport (Fig. 3C, Supplementary Data Tables S3 andS4).

**Figure 4:**
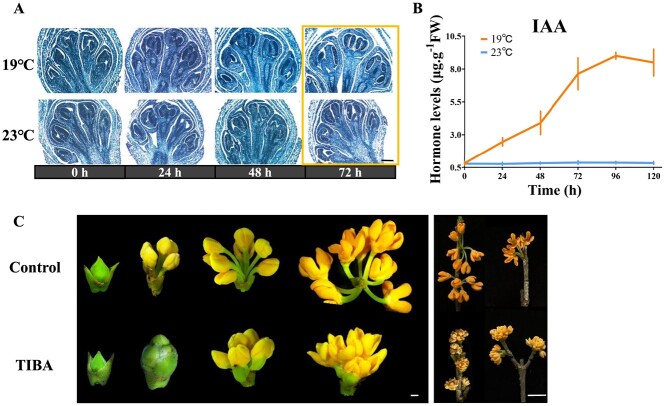
Auxin's effect on *O. fragrans* flower opening initiation. **A** Longitudinal structural sections of floral buds treated at 19°C (top) and 23°C (bottom) for 24, 48 and 72 h. Scale = 1 μm. **B** Endogenous IAA levels in floral buds treated at 19 and 23°C for 24, 48, 72, 96, and 120 h. **C** Floral buds' phenotype with (top)/without (bottom) auxin inhibitor (2,3,5-Triiodobenzoic acid, TIBA) after 2, 8 10, and 11 days. Scales = 1 mm (left), 1 cm (right).

**Figure 5:**
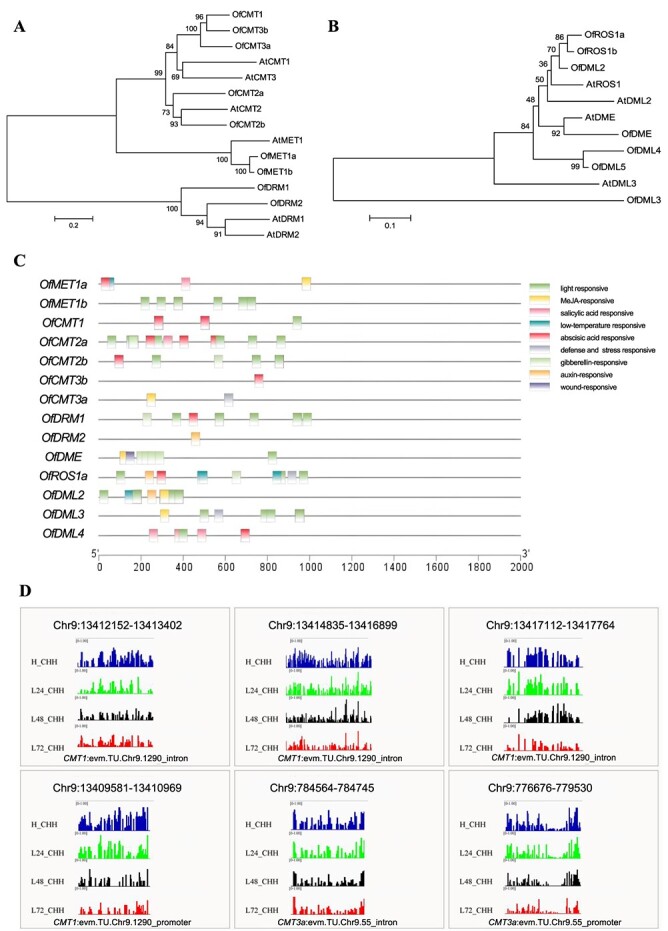
Sequence analysis of DNA methyltransferase and DNA demethylase genes in *O. fragrans*. **A**, **B** Phylogenetic analysis of DNA methyltransferase (**A**) and DNA demethylase (**B**) in *O. fragrans*. **C***Cis*-acting elements in promoters of DNA methyltransferase genes and DNA demethylase genes. **D** IGV snapshots of DNA methylases containing hypomethylated DMRs in *Osmanthus* buds with diverse durations of 19°C treatment.

### Temperature-dependent accumulation of endogenous auxin elongated peduncles in floral buds

To further elucidate the influence of temperature on the initiation of floral bud opening, we examined the longitudinal ultrastructure of plants subjected to 19 and 23°C treatments using paraffin sections. As depicted in Fig. 4, no notable differences were observed in the ultrastructure of floral buds treated at 19°C for either 24 or 48 h compared with those consistently treated at 23°C (Fig. 4A, Supplementary Data Table S5), aligning with the previously observed phenotypes (Fig. 1D). The rapid elongation of the peduncle, resulting in floral bract cracking, occurred at the 72nd hour following the 19°C treatments (Fig. 4A). This timeframe of 0–72 h was also identified as a stage when temperature no longer restricted the flower opening process (Fig. 1D and E). Thus, it suggested that peduncle elongation was a notable trait associated with the initiation of flower opening in *O. fragrans*. Auxins are among the most essential plant hormones; as growth regulators they have multiple roles in plant development, such as cellular elongation and expansion [46, 47]. The contents of indole-3-acetic acid (IAA), the most common auxin-class phytohormone, in the floral buds of S1–S2 (0–120 h) exposed to 19°C were examined in *O. fragrans*. The findings demonstrated that under ALT endogenous IAA content accumulated in floral buds compared with those that remained closed at 23°C (Fig. 4B). To explore the influence of auxin on flower opening, a polar auxin transport inhibitor, 500 mg/l of 2,3,5-triiodobenzoic acid (TIBA), was sprayed on flowering branches with mature floral buds of *O. fragrans* at 19°C (Fig. 4C). Although the flower buds treated with TIBA still exhibited opening, the peduncle length of fully bloomed *O. fragrans* was notably reduced, which meant that the peduncle’s inability to elongate was attributable to the lack of endogenous auxin supply to the floral buds (Fig. 4C, Supplementary Data Table S6).

In summary, the accumulation of endogenous auxin induced by low ambient temperatures significantly promoted peduncle elongation, which emerged as a critical trigger for the initiation of flower opening in *O. fragrans*.

### Association analysis of DNA methylation status and gene expression levels

The Venn diagram in Supplementary Data Fig. S5A illustrates how DMRs within the gene body and promoter regions impact DEGs. In the comparisons of L24 versus H, the proportions of upregulated and downregulated DEGs associated with hypomethylated DMR genes were 0.9% (60) and 1.3% (90), respectively. For L48 versus H, these proportions were 1.8% (161) and 1.9% (171), and for L72 versus H, they were 2.0% (215) and 2.3% (246) (Supplementary Data Fig. S5A). Similarly, in the same comparisons, the proportions of overlapping upregulated and downregulated DEGs with hypo-DMR promoter genes were 1.6% (111) and 1.3% (93) for L24 versus H, 2.7% (250) and 2.4% (215) for L48 versus H, and 3.3% (356) and 2.8% (297) for L72 versus H (Supplementary Data Fig. S5A).

The increased occurrence of hypo-DMRs in the genome with extended exposure to 19°C indicated a regulatory influence of ALT on reducing methylation levels. To explore the correlation between DNA methylation levels and transcriptome profiles, genes were categorized into four groups based on their expression levels: unexpressed (none, fpkm < 1), low expression (1 ≤ fpkm < 25% × fpkm), middle expression (25% × fpkm ≤ fpkm < 75% × fpkm), and high expression (fpkm ≥ 75% × fpkm) (Supplementary Data Fig. S5B). Genes with no expression exhibited the highest DNA methylation levels in all contexts downstream of the gene body, as well as in the CHG and CHH contexts within the gene body, and in the CG and CHG contexts upstream of the gene body (Supplementary Data Fig. S5B). The methylation levels within the gene body in the CHG and CHH contexts were discovered to exert a positive regulatory influence on the expression level (Supplementary Data Fig. S5B). However, the methylation status of the promoter regions in the CHH contexts did not exhibit a notable correlation with transcriptional activity (Supplementary Data Fig. S5B).

We subsequently categorized the methylated genes into five groups according to their levels of methylation in the promoter and gene body regions respectively (first, methylation level < 20%; second, 20% < methylation level < 40%; third, 40% < methylation level < 60%; fourth, 60% < methylation level < 80%; and fifth, methylation level > 80%). Supplementary Data Fig. S5C reveals that gene body regions displaying the highest levels of methylation exhibited the lowest expression levels in the CHG and CHH contexts. Furthermore, genes exhibiting the highest levels of methylation in their promoters displayed reduced gene expression in the CG and CHG contexts, while demonstrating elevated gene expression in the CHH context (Supplementary Data Fig. S5C).

### Association analysis of DNA transcriptome profiling and methylation status of genes involved in flower opening

To investigate the relationship between DNA methylation and the onset of sweet osmanthus flower opening, we examined the distribution of DMRs within gene regions linked to auxin signal transduction, cell expansion and elongation, sucrose synthesis, and temperature signal response during the temperature-responsive phase preceding flower opening. Firstly, we focused on DEGs associated with auxin signal transduction, including six transcripts encoding auxin-responsive proteins (OfIAAs), one transcript encoding auxin-induced protein (OfAUX22), one transcript encoding transport inhibitor response protein 1 (OfTIR1), seven transcripts encoding auxin response factors (OfARFs), seven transcripts encoding small auxin upregulated RNAs (OfSAURs), and one transcript encoding an auxin transporter-like protein (OfLAX3a) (Supplementary Data Table S7). These genes exhibited at least one DMR within their respective genomic functional regions at the 24-, 48-, and 72-h time points following the 19°C treatment (Supplementary Data Fig. S6A, Supplementary Data Tables S5 and S8–S10). Itegrative Genomics Viewer (IGV) snapshots of representative hypo- and hyper-DMRs in floral buds induced to open revealed that many loci associated with auxin response and transport displayed lower methylation levels in the floral buds of L24, L48, and L72 compared with those of H. This trend was particularly evident in the floral buds of L48 and L72 within the CHH contexts, where a higher count and magnitude of hypo-DMRs were observed in comparison with the H buds (Supplementary Data Fig. S6). The converse outcome was observed in the intron region of *OfIAA31* for the L48 floral buds, and similarly in the same promoter regions of *OfAUX22* for both the L48 and L72 floral buds (Supplementary Data Fig. S6B). Among the set of 21 hypo-DMR-associated DEGs, 15 genes, including *OfARF3*, *OfARF4*, *OfARF8*, *OfARF17*, *OfARF19*, *OfSAUR50a*, *OfSAUR50b*, *OfSAUR50d*, *OfSAUR61*, *OfSAUR72*, *OfIAA7*, *OfIAA8*, *OfIAA11*, *OfIAA34*, and *OfLAX3a*, demonstrated an upregulated expression profile upon exposure to the 19°C treatment (Supplementary Data Table S7). This finding suggests a potential negative correlation between methylation levels and transcript abundance in genes associated with the auxin signal transduction pathway.

Within 48–72 h after induction at 19°C, the inflorescence exhibited enlargement along with noticeable peduncle elongation (Fig. 4). The expansion growth of petals, facilitated by cell-wall relaxation, involved key proteins such as expansins and xyloglucan endotransglycosylase/hydrolases (XTHs), which reside in the apoplast [48, 49]. Six *EXP*s (*OfEXPB3*, *OfEXPA8a*, *OfEXPA8b*, *OfEXP15a*, *OfEXP15b*, and *OfEXPB16*) and two *XTHs* (*OfXTH2* and *OfXTH10*) exhibited transcriptional activation at 24 h after the initiation of the 19°C treatment (Supplementary Data Table S7). Notably, *OfEXP8A* was the sole gene to display hyper-DMRs in the CG contexts across both its intron and exon regions in response to temperature change, whereas the remaining *OfEXPs* and *OfXTHs* were marked by at least one hypo-DMR, predominantly located in their promoter regions (Supplementary Data Fig. S7). Similar trends were observed for genes associated with carbohydrate metabolism. The pattern depicted in Supplementary Data Fig. S8 illustrates that the sucrose content in floral buds initially decreased during the initial 24 h of exposure to 19°C, followed by a steady and substantial increase. This temporal pattern coincided with the noticeable hypo-DMRs identified in genes encoding sucrose synthase (*OfSUS1* and *OfSUS6*) within their promoter regions. These DNA methylation changes corresponded well with the observed marked increase in their expression levels following the 19°C induction (Supplementary Data Figs S7 andS8, Supplementary Data Table S7).

Ethylene, a key plant hormone, significantly affects growth, development, and responses to environmental cues like low temperatures [50–52]. We examined 1-aminocyclopropane-1-carboxylic acid (ACC), a direct ethylene precursor, in *O. fragrans* floral buds at 19°C. Our findings revealed a remarkable upward trend, particularly a significant increase after 24 h of treatment (Supplementary Data Fig. S8). Eleven ERF/AP2 transcription factors (*OfAIL1*, *OfCRF2*, *OfTOE3*, *OfERF2*, *OfERF3*, *OfERF5*, *OfERF11*, *OfERF81*, *OfERF92*, *OfERF110*, *OfERF118*) and ethylene response 1 (*OfETR1*), had hypo-DMRs and distinct expression under 19°C (Supplementary Data Tables S8–S10). Similar to the flower opening-related genes mentioned above, These DMRs primarily resided in CHH-context promoter regions. Most genes with hypo-DMRs (8 of 12) showed upregulated expression (Supplementary Data Tables S7–S10). In contrast, after 48 h of induction at 19°C, the expression of *OfERF81*, which harbored hyper-DMRs in the CG contexts of the promoter regions, also displayed a significant augmentation (Supplementary Data Tables S7–S10). Furthermore, we also identified DEGs linked to DMRs, involved in temperature perception, response, and signal pathways [53–55]. These included genes for 10 heat shock proteins (*OfHSP*s: *OfHSP70a*, *OfHSP70b*, *OfHSP72*, *OfHSP22a*, *OfHSP11*, *OfHSP181*, *OfHSP22b*, *OfHSP70c*, *OfHSP70d*, *OfHSP70e*), 7 calcium-dependent protein kinases (*OfCDPK*s: *OfCDPK1*, *OfCDPK9*, *OfCDPK11*, *OfCDPK13*, *OfCDPK16*, *OfCDPK17*, *OfCDPK29*), and 8 mitogen-activated protein kinases (*OfMAPK*s: *OfM3K1*, *OfMMK2a*, *OfMMK2b*, *OfM2K6*, *OfMPK19*, *OfM2K5*, *OfMPK16*, *OfM3K18*) (Supplementary Data Tables S7–S10). Notably, among these only *OfM2K6*, *OfMMK2b*, and *OfCDPK1* contained hyper-DMRs, whereas the remaining loci all exhibited hypo-DMRs under 19°C treatments compared with floral buds of H in *O. fragrans* (Supplementary Data Tables S7–S10).

**Figure 6:**
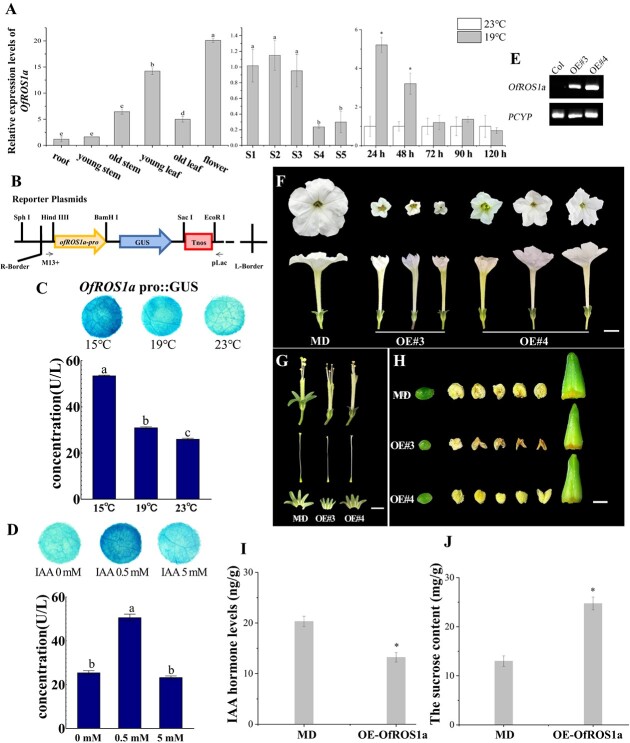
Effects of *OfROS1a* overexpression on flowers in *P. hybrida* cv. ‘Mitchell Diploid’ (MD). **A** Expression of *OfROS1a* in roots, young and old stems, young and old leaves, flowers (left), in flowers at five stages of flower opening (middle), and in flowers within 120 h of S1–S2 stage (right). **B***OfROS1a* promoter was linked to pCAMBIA1300-GUS, with the CaMV 35S promoter removed. **C**, **D** GUS activity assay by transiently transforming into leaves of *N. benthamiana* with *OfROS1a*pro::GUS, at 15, 19, and 23°C (**C**) under IAA concentrations of 0, 0.5, and 5 mM (**D**). **E** Results of semi-quantitative RT–PCR of *OfROS1a* in MD and *OfROS1a*-overexpression petunia. **F** Top views (top) and side views (bottom) of *OfROS1a*-overexpressing opened flowers (middle and right) and control flowers (left) in petunia. Scale bar = 1 cm. **G** Unpollinated ovary (left), stigma (middle), and anthers (right) of *OfROS1a*-overexpressing (middle and bottom) and control (top) plants. Scale bar = 1 mm. **H** Stamens (top), pistils (middle), and sepals (bottom) of controls (left) and *OfROS1a*-overexpressing petunia (middle and right). Scale bar = 1 cm. **I**, **J** Auxin levels (**I**) and sucrose contents (**J**) in controls and *OfROS1a*-overexpressing petunia.

### Effect of ambient low temperature treatment on the abundance and DNA methylation of *DNA methyltransferases* and *DNA demethylases* for floral buds

DNA methylation is governed by DNA methyltransferases and demethylases. We aligned orthologs of *Arabidopsis* DNA methyltransferase and demethylase genes in the *O. fragrans* genome. We identified nine DNA methyltransferase genes (*OfMET1a*, *OfMTE1b*, *OfCMT1*, *OfCMT2a*, *OfCMT2b*, *OfCMT3a*, *OfCMT3b*, *OfDRM1*, *OfDRM2*) and 7 DNA demethylase genes (*OfDME*, *OfROS1a*, *OfROS1b*, *OfDML2*, *OfDML3*, *OfDML4*, *OfDML5*) through phylogenetic analysis (Fig. 5A and B, Supplementary Data Table S11). Promoter analysis of these genes unveiled low-temperature-responsive elements in *OfMET1a, OfROS1a*, and *OfDML2* and auxin-responsive elements in *OfDRM2*, *OfROS1a*, and *OfDML2* using the online tool PlantCARE (Fig. 5C). Additionally, the promoter of *OfROS1a* revealed a multitude of other hormone-responsive elements, including those that respond to methyl jasmonate (MeJA), abscisic acid (ABA), and gibberellins (GAs). After 19°C treatments, DNA demethylase genes, *OfDME*, *OfROS1a*, and *OfDML3*, were activated, while the DNA methyltransferase gene *OfCMT2b* was suppressed (Supplementary Data Fig. S9, Supplementary Data Table S11), implying a great possibility that these genes were involved in the hypomethylation regulation of the flower opening initiation in *O. fragrans*. *OfDML2* showed no significant difference during diverse treatment times (Supplementary Data Fig. S9, Supplementary Data Table S11). Interestingly, hypo-DMRs in CHH contexts within intron and promoter regions of *OfCMT1* and *OfCMT3a* were observed after 19°C treatment, with their upregulated transcription peaking at 72 h (Fig. 5D, Supplementary Data Fig. S9, Supplementary Data Table S11), which suggests a potential scenario where the lower methylation could potentially exert a positive regulatory effect on DNA methyltransferase genes, consequently triggering a negative feedback loop of hypomethylation in *O. fragrans* (Fig. 5D, Supplementary Data Fig. S9).

### Overexpression of *OfROS1a* inhibited accumulation of endogenous auxin and increased the concentration of sucrose

We were interested in whether *OfROS1a* was involved in the ALT response to initiate flower opening after floral bud maturation in *O. fragrans*. Thus, the expression of *OfROS1a* in different plant leaves, stems, and flower organs at different developmental stages was examined by quantitative RT–PCR (qPCR) firstly. *OfROS1a* exhibited high transcription in young leaves and flower organs. During flower opening, the expression of *OfROS1a* peaked at S1–S3 and subsequently declined from S4 (Fig. 6A). After 24 h at 19°C, the expression of *OfROS1a* was notably increased, remaining higher than in mature floral buds at 23°C even after 48 h, consistent with the *OfROS1a* fpkm profile following the 19°C treatments mentioned above (Fig. 6A, Supplementary Data Fig.S9). To assess the responsiveness of *OfROS1a* to temperature and auxin signals, we inserted the *OfROS1a* promoter DNA into a pCAMBIA1300-GUS vector and transiently transformed it into *Nicotiana benthamiana* (Fig. 6B). *β*-Glucuronidase (GUS) activity assays revealed reduced *OfROS1a*pro::GUS activity at higher temperatures (23°C) compared with 15 and 19°C (Fig. 6C). Additionally, exogenous auxin introduced at 23°C enhanced GUS activity driven by *OfROS1a* promoters, with 0.5 mM auxin showing the most prominent effect (Fig. 6D). However, 5 mM auxin had no significant impact on promoter activity compared with the control, yet it did suppress activity compared with the 0.5 mM auxin treatment (Fig. 6D). These results indicated that low temperatures and low auxin concentrations induced *OfROS1a* promoter activity, whereas high auxin concentrations repressed it.

We proceeded to overexpress *OfROS1a* in *Petunia hybrida* and assessed its biological function using two overexpression lines (OE#3 and OE#4) using a semi-quantitative RT–PCR assay (Fig. 6E). The 35S:*OfROS1a* overexpression lines demonstrated significant effects on petal unfolding and the development of floral organs, including corollas, sepals, pistils, stigmas, ovaries, stamens, and anthers (Fig. 6F–H). Unexpectedly, overexpression of *OfROS1a* resulted in a reduction in corolla size, as well as pistil and stamen length in petunia, possibly due to the decreased concentration of IAA in petunia (Fig. 6F–I, Supplementary Data Table S12). This indicated that overexpressed *OfROS1a* under the CaMV 35S promoter acted as a signal for negative feedback in endogenous auxin accumulation. Moreover, *OfROS1a* increased the sucrose concentration in petunia corollas, consistent with the increased sucrose contents observed during the 19°C response stages prior to flower opening in *O. fragrans* (Fig. 6J, Supplementary Data Fig.S8). To evaluate differential gene expression in *OfROS1a*-overexpressing petunia compared with the control via mRNA-seq, a volcano plot highlighted significant changes in gene distribution, revealing 763 upregulated genes and 1138 downregulated genes [|log2(FoldChange)| > 1 and padj  < 0.05] (Supplementary Data Fig. S10A). GO analysis showed that the upregulated genes in *OfROS1a*-overexpressing petunia were primarily involved in chemical response, auxin response, and transmembrane transporter activities (Supplementary Data Fig. S10B). In contrast, the differentially downregulated genes were significantly enriched in the carbohydrate metabolism process (Supplementary Data Fig. S10C). Furthermore, KEGG pathway analysis of DEGs resulting from *OfROS1a* overexpression revealed an enrichment of the spliceosome and RNA degradation pathways among upregulated DEGs (Supplementary Data Fig. S10D). Likewise, the pathways of starch and sucrose metabolism and tryptophan metabolism exhibited enrichment among downregulated DEGs (Supplementary Data Fig. S10E). To summarize, these results strongly correlated *OfROS1a* with signaling, RNA processing, and carbohydrate metabolism.

## Discussion

DNA methylation, as a fundamental epigenetic modification, plays a crucial role in various biological processes by dynamically regulating establishment, maintenance, and active removal mechanisms [56]. In this study, we uncovered a DNA hypomethylated regulatory process that takes place during temperature-dependent flower opening in *O. fragrans*. We also attempted to analyze the mechanisms underlying the dynamic response of DNA methylation to ALT and discuss the significance of DNA hypomethylation regulation in triggering flower opening in *O. fragrans.*

### DNA hypomethylation regulation of flower opening initiation is temperature dependent in *O. fragrans*

In our study, we investigated how unsuitable ambient temperatures (≥23°C) disrupted the flowering process in *O. fragrans* once floral bud development reached maturity, leading to failed flower opening (Fig. 1). A short period of lower temperatures (<23°C) for a few days acts as a signal to initiate flower opening, allowing the flowering process to continue in *O. fragrans* (Fig. 1C). This phenomenon differed from some temperature-sensitive flowering plants that continue their flower opening process with a prolonged chilling requirement lasting several months, even after floral meristem differentiation, like *Prunus mume*, *Chimonanthus praecox*, and *Prunus persica* [18, 57, 58]. These plants exhibited a prolonged influence of cold temperature, whereas our study identified that 19°C quickly triggered flower opening in *O. fragrans* as the optimal ambient temperature. Moreover, we identified that the interval encompassing the floral buds of S1 through to the stage when the bracts of the outermost enveloping inflorescences had preliminarily dehisced represented a pivotal phase of ALT responsiveness in determining flower opening (Fig. 1D).

DNA methylation plays a vital role in temperature-dependent biological processes, with distinct patterns modulated by varying temperatures. In peach, DNA hypermethylation during chilling ensured flower development programming free from residual *DAM* inhibition [18]. Moreover, post-harvest peaches stored at 16°C exhibited low DNA methylation levels in the flesh, resulting in anthocyanin accumulation, a phenomenon not observed at or below 12°C [14]. Low temperatures induced cucumber femaleness through genome-wide reductions in mC frequency and average DNA methylation levels [59]. Similarly, in *O. fragrans* the ALT-dependent flowering phase led to a global decrease in DNA methylation across CG, CHG, and CHH contexts (Fig. 2). DNA-hypomethylated regulation was also reported to influence flower expansion and opening during S3–S5 in *O. fragrans* [33]*.* Our findings further reveal a pronounced decrease in CHH methylation levels, accompanied by a significant occurrence of DMRs in the CHH context. This underscored the substantial role of CHH methylation dynamics in regulating DNA hypomethylation during the ALT-responsive flower opening phase in *O. fragrans*. Notably, CHH demethylation emerges as the primary response to low-temperature treatment [18, 59], contrasting with the prevalence of alterations in CG methylation status due to bacterial infection [60]. Additionally, salt stress predominantly induces DNA demethylation in CG contexts, accompanied by a rapid demethylation response in CHG contexts [61].

**Figure 7:**
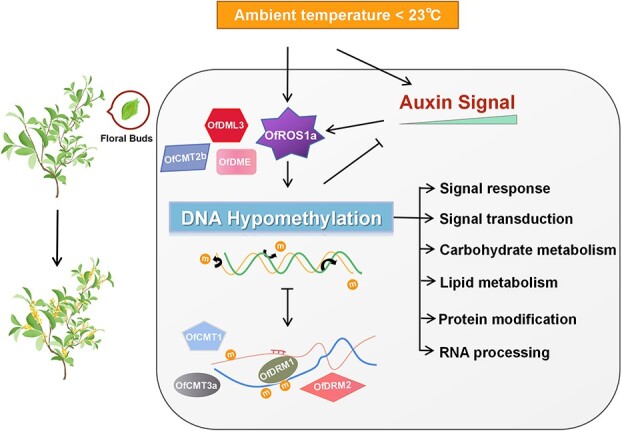
Active DNA hypomethylation regulation of ambient low temperature responsive phase of flower opening in *Osmanthus fragrans* via DNA demethylases and DNA methylases.

Ethylene, a gaseous plant hormone, significantly influences growth, development, and responses to environmental cues, including low temperatures [51]. It promoted flower opening in *Rosa hybrida* through asymmetric petal base growth [10]. Key ethylene-related genes like *ERF/AP2*s and *ETR1* participated in plant responses to low temperatures [62–64]. In *O. fragrans*, ACC accumulation induced by ALT suggested that ethylene signaling responded to ambient temperature changes, activating floral bud opening (Supplementary Data Fig. S8). Eleven *ERF/AP2* transcription factors and one *ETR1*, with hypo-DMRs mainly in CHH contexts of promoters, exhibited three transcriptional downregulations and nine upregulations (Supplementary Data Tables S7–S10). Despite CHH promoter methylation’s weak correlation with transcription, possibly due to lower CHH levels compared with CG and CHG contexts, a majority of genes in the ethylene pathway, regulated by DNA demethylation, became active during ALT before flower opening (Supplementary Data Table S7). DNA hypomethylation also regulated flower senescence via the ethylene pathway in *O. fragrans* [33]. RNA-seq data identified EDGs related to plant perception and temperature responses, including 10 *HSP*s, 6 *CDPK*s, and 6 *MAPK*s with hypo-DMRs (Supplementary Data Tables S7–S10) [53, 65]. This discovery showed that DNA hypomethylation-mediated regulation activated or suppressed pathways for low- and high-temperature responses. Unlike *Hibiscus cannabinus*, CDPK and MAPK pathways, influenced by DNA methylation, were significant for cytoplasmic male sterility [66]. In poplar, CHH hypomethylation induced CDPK24, a participant in immune responses to pathogens [67]. However, DNA methylation’s regulatory role in MAPK, CDPK pathways, and HSPs for flowering remains uncertain.

The results mentioned above demonstrated that ALT induced the DNA hypomethylation responsible for regulating the initiation of flower opening in *O. fragrans*. These findings greatly enhance our comprehension of the mechanisms underlying ALT-induced flower opening.

### Active DNA demethylation contributes most to the DNA hypomethylation regulation of ambient low temperature response in flower opening

In plants, DNA methyltransferases and demethylases modulate DNA methylation status, often at the transcriptional level, responding to various environmental cues [14, 56]. In *Citrus sinensis*, cold-induced *DML1* expression in highly pigmented fruit areas may contribute to orange’s blood-red coloration [68]. Similarly, apples show increased *ROS1* transcription under low temperatures, leading to anthocyanin buildup [27]. In our study, both 15 and 19°C ALT exposure activated *OfROS1a* expression, which had low-temperature-responsive elements in its promoters (Fig. 5C). This activation did influence the flowering process in *O. fragrans* (Fig. 6). Interestingly, *OfROS1a*pro::GUS activity was higher at 15 than 19°C, unlike flower opening rates under ALT treatments (Fig. 6C). This suggests that the combined effects of DNA methyltransferases and demethylases drive the ALT response via DNA hypomethylation. *OfDME* and *OfDML3* expression increased, while all DNA methylases except *OfCMT2b* showed elevated expression at 19°C as well (Supplementary Data Fig. S9), differing from the process of DNA hypomethylation regulation associated with petal unfolding in *O. fragrans* [33]. Furthermore, *OfCMT1* and *OfCMT3a* were notably transcriptionally activated, possibly controlled by DNA demethylation, after 48 h at 19°C (Fig. 5D). This implies that plants may have a mechanism for finely orchestrating DNA hypomethylation dynamics and maintaining homeostasis in the DNA methylation landscape while regulating flowering across different stages (Fig. 7). Through evolution, flowering plants have developed at least three epigenetic homeostasis mechanisms that ensure the high-fidelity maintenance of DNA methylation via positive feedback loops [69]. In mammals, factors including methyl-DNA binding protein Kaiso contribute to DNA methylation dynamics [70]. These findings collectively suggest the potential involvement of multiple DNA methyltransferases and demethylases in the ALT-responsive flower opening phase, underscoring the significant role of active DNA demethylation in this process in *O. fragrans*.

### Regulation of DNA hypomethylation induced RNA-directed DNA methylation catalyzed by OfDRM1 and OfDRM2 during the later ALT response phase of flower opening

In plants, *de novo* DNA methylation occurs via the RNA-directed DNA methylation (RdDM) pathway, reliant on small interfering RNAs (siRNAs) and scaffold RNAs [56]. RdDM is crucial for inducing DNA methylation alterations, observed in temperature-based sex determination in cucumber [59], and epigenetic control of thermal-responsive floral bud dormancy in peach [18]. Reducing 24-nucleotide siRNA levels that guide *de novo* methylation resulted in lowered DNA methylation caused by low temperatures, particularly in the CHH sequence context of transposable elements [71]. These outcomes align with our findings, which showed a significant methylation reduction in the CHH context of genomic repeat regions, implying a potential compensatory mechanism through unknown regulatory processes to partially counteract the decrease in *de novo* methylation (Fig. 2A). Our research showed increased RNA processing during the ALT-responsive flower opening phase (Supplementary Data Tables S3 andS4). Overexpressed *OfROS1a* activated RNA degradation and splicing-associated genes (Supplementary Data Fig. S10). Additionally, slight transcriptional activation of *OfDRM2* and *OfDRM1*, mainly governing *de novo* DNA methylation, was observed (Supplementary Data Fig. S9). Given that ALT-induced DNA hypomethylation was mainly driven by active DNA demethylation, it implied that *de novo* DNA methylation’s later-stage activation could be involved in DNA methylation homeostasis, aligning with our previous discussions.

### Temperature-dependent auxin accumulation modulates the pathways of auxin signal transduction through dynamic DNA methylation to induce flower opening

Our study revealed a pivotal discovery: temperature-induced IAA accumulation, driving peduncle elongation, plays a crucial role in initiating flower opening in *O. fragrans* (Fig. 4). In iris (*Iris × hollandica*), flower opening required pedicel and ovary elongation, both governed by endogenous auxins [72]. Similarly, water lilies’ flower opening and closure rhythm were auxin-controlled [73]. These instances underscore auxin’s significance in pedicel elongation and petal movement. DNA hypomethylation is implicated in regulating auxin signal transduction pathways during vital biological events like vegetative growth, petal unfurling, and fruit ripening [26, 33, 74]. Our research findings parallel this, identifying DNA hypomethylation’s role in the auxin signaling pathway during the ALT-responsive flower opening phase (Supplementary Data Fig. S6). However, the intricate interplay between auxin signaling and DNA hypomethylation during flowering remains unclear. In pepper fruit ripening, exogenous IAA distinctly upregulated *CaMET1* and *CaCMT3* expression [26]. Similarly, auxin treatment upregulated DNA methyltransferase genes like *DRM1*, *DRM2*, and *MET1*, while downregulating demethylase genes like *ROS1*, *DME*, and *DML* [75]. Notably, we observed that the promoters of *OfROS1a*, containing auxin-responsive elements, were significantly activated only by low-concentration exogenous IAA, not high concentrations (Fig. 6D). This observation may partly explain why *OfROS1a*’s transcription peaked after 24 h of 19°C treatment, subsequently declining in *O. fragrans* floral buds (Supplementary Data Fig. S9). Moreover, DNA hypomethylation reciprocally influences auxin signaling. *CaMET1* silencing-induced DNA hypomethylation suppressed auxin signaling and reduced auxin levels in pepper fruit [26]. Likewise, *drm1-drm2-cmt3* mutants in *A. thaliana* exhibited auxin deficiency-related morphological abnormalities [74]. Thus, passive DNA demethylation due to the loss of DNA methylase activity can inhibit auxin signaling. Our study elucidated a negative feedback mechanism in auxin signaling triggered by active DNA demethylation through *OfROS1a* overexpression (Fig. 6J). Additionally, *OfROS1* was found to enhance the transcription of genes involved in the auxin signal transduction pathway through its overexpression (Supplementary Data Fig. S10B).

In summary, our study unveils a feedback loop involving active DNA hypomethylation status that mediates auxin-regulated signal transduction during the ALT-responsive flower opening phase (Fig. 7).

### DNA hypomethylation regulated carbohydrate and lipid metabolism during the ambient low-temperature-responsive stage of flower opening

Carbohydrate metabolism significantly influences plant flower opening. Petal cells in numerous species stored ample starch, which converts to glucose and fructose just before opening [76]. Flower opening often involved stored carbohydrate mobilization and/or sucrose import [77]. In *Sandersonia*, petals accumulated glucose and fructose by absorbing sucrose from the apoplast prior to opening [78]. Lipid metabolism also played a role, as disrupted lipid metabolism impairs flower opening in *A. thaliana* [79]. Flowers treated with Promalin exhibit delayed opening accompanied by transient lipid hydroperoxide elevation during lily flower opening [80]. Our study revealed ALT-induced hypo-DMRs associated with EDGs, enriched in carbohydrate and lipid metabolism (Fig. 3). This indicates that DNA hypomethylation-mediated regulation extended to carbohydrate and lipid metabolism during the ALT-responsive flower opening phase. Additionally, we observed sucrose accumulation during the preparatory phase of flower opening, involving *OfROS1a*-induced DNA hypomethylation in sucrose synthesis (Fig. 6, Supplementary Data Fig. S8). DNA methylation dynamics from *OfROS1a* overexpression also regulate other carbohydrates (Supplementary Data Fig. S10), as seen in apple floral buds [81]. However, lipid metabolism remained unaffected by OfROS1a, suggesting alternative DNA methyltransferases and demethylases’ involvement (Supplementary Data Fig. S10).

In this paper, we provide evidence supporting the involvement of DNA demethylases, particularly OfROS1a, in orchestrating DNA methylation dynamics that governed the ALT-induced flower opening process. We also propose that the upregulation of DNA methylase genes during the rapid ALT response helps maintain the fidelity and dynamic homeostasis of DNA methylation as the plant transitions to the next flowering stage. Additionally, our findings shed light on active DNA hypomethylation-mediated auxin signaling modulation and carbohydrate and lipid metabolism, as well as the rapid transcriptional changes of genes associated with temperature response and flower development, all of which occurred within a short time frame. Further investigation into the detailed mechanisms of active DNA hypomethylation during temperature-dependent flower opening warrants in-depth exploration. While similar to most woody plants, the incipient transgenic technology, hindered by the complexity of the genome, poses challenges for research on *O. fragrans*. The highly efficient transient gene expression protocols we previously established for *Osmanthus* can be a valuable tool for molecular regulation validation [82]. Furthermore, as biotechnology continues to advance, molecular research on *Osmanthus* will be expected to make greater progress in the future. Our research contributes to the growing body of knowledge on the complex regulatory networks involved in plant development and environmental adaptation, laying a foundation for the controlled cultivation of *O. fragrans* and molecular breeding for temperature-sensitive plants during their flowering phases.

## Materials and methods

### Plant materials

The ‘Yanhong’ cultivar of *O. fragrans* was sourced from the *Osmanthus* Germplasm Resource Preservation Center at Zhejiang Agriculture and Forestry University in Hangzhou, China. To maintain uniform growth conditions, all plant materials were grown in climate chambers set at a temperature of 25 ± 2°C, with a 12-h light and 12-h dark cycle, and a relative humidity level of 60%. Diverse organs, encompassing young and mature leaves, young and mature stems, and roots, were collected. Floral buds subjected to 24 (L24), 48 (L48), and 72 (L72) h of treatment in a 19°C climatic chamber were collected post-maturation, while floral buds at 19°C for 0 h (H) served as the control. Our experiments were conducted with a total of three biological replicates, each utilizing independently collected and extracted tissues.

### Analysis of flowering index among across various ambient temperature treatments

*Osmanthus fragrans* plants bearing fully mature floral buds in the 25°C climate chamber were transferred to different climate chambers with a temperature gradient ranging from 17 to 27°C. The flower opening stages of *O. fragrans* were classified into five distinct phases: stage 1 (S1) was designated with a count of 0; stage 2 (S2) corresponded to a count of 1; stage 3 (S3) denoted a count of 2; stage 4 (S4) signified a count of 3; and stage 5 (S5) represented a count of 4. Cumulative counts of treated floral buds, each paired with the respective stage count, were calculated every 24 h, and then weighted averaging was applied to generate comprehensive statistical analysis about the floral buds. All experiments were performed employing 15 biological replicates of plants, sourced from independently collected and extracted tissues.

### Whole-genome bisulfite sequencing and analysis

DNA quality and quantity assessments were as follows: DNA purity was assessed using the NanoPhotometer^®^ spectrophotometer from Implen (CA, USA), while DNA concentration was quantified using the Qubit^®^ DNA Assay Kit on the Qubit^®^ 2.0 Fluorometer from Life Technologies (CA, USA). DNA quality was confirmed via agarose gel electrophoresis. Following bisulfite treatment with the EZ DNA Methylation-Gold™ Kit from Zymo Research, Novogene Corporation (Beijing, China) conducted library construction. The library quality was assessed using the Agilent Bioanalyzer 2100 system. Subsequently, paired-end sequencing of the samples was performed using the Illumina platform from Illumina (CA, USA). For DMR identification, we used DSS software [83]. We performed GO enrichment analysis of DMR-associated genes using the GOseq R package [84], identifying significantly enriched GO terms with a *P*-value <0.05. Additionally, we conducted statistical enrichment analysis of DMR-associated genes in KEGG pathways using the KOBAS software, with significance set at a corrected *P*-value of 0.05 and an absolute fold change of 2 [85].

### RNA extraction and RNA-seq analysis

We assessed RNA integrity using the RNA Nano 6000 Assay Kit on the Agilent Bioanalyzer 2100 system from Agilent Technologies (CA, USA). Afterwards, the index-coded samples were clustered with TruSeq PE Cluster Kit v3-cBot-HS from Illumina. Subsequently, the library preparations were subsequently sequenced on an Illumina Novaseq platform.

We utilized the reference genomes for ‘Liuyejingui’ obtained from *O. fragrans* (https://www.ncbi.nlm.nih.gov/genome/?term=txid93977[orgn]) and *P. hybrida* (https://solgenomics.net/ftp/genomes/Petunia_axillaris/assembly/Petunia_axillaris_v1.6.2_genome.fasta). The reference genome index was constructed using HISAT2 v2.0.5 and paired-end clean reads were aligned to the reference genome employing HISAT2 v2.0.5. Subsequently, the number of reads mapped to each gene was counted using FeatureCounts v1.5.0-p3. Differential expression analysis between the two groups was performed using the DESeq2 R package (1.20.0), designating genes with an adjusted *P*-value <0.05 as differentially expressed. We set the thresholds for significant differential expression at a corrected *P*-value of 0.05 and an absolute fold change of 2. The RNA-seq analysis was conducted by the experts at Novogene Bioinformatics.

### Measurements of hormone and sucrose contents

Floral buds (1.5 g fresh weight) from *O. fragrans* ‘Yanhong’ were collected for extraction using a methanol mixture and an internal standard. The phytohormones were detected and analyzed using the ExionLC UPLC system from AB Sciex (USA), which was equipped with an Acquity UPLC CSH C18 column (1.7 μm, 2.1 × 150 mm, Waters), as previously outlined. The analysis was conducted with a total of three biological replicates [86].

For each biological replicate, 0.5 g of sucrose (dry weight) was extracted from floral bud samples, following the previous description [87]. Prior to HPLC analysis, the sample was filtered through a 0.22 μm syringe filter. Sucrose analysis was carried out using an HPLC system from Shimadzu (Kyoto, Japan). The separation was performed on an Aminex HPX-87P column (Bio-Rad, USA) measuring 300 × 7.8 mm with a particle size of 9 μm.

### Auxin transporter inhibiter treatment

Twelve healthy *O. fragrans* ‘Yanhong’ plants were selected and evenly divided into two groups, with six replicates in each group. When the floral buds were fully matured at 25°C, six *Osmanthus* plants were subjected to TIBA treatment at 19°C by spraying with a solution containing 1% ethanol and 500 mg/l TIBA (Yanaye Bio-Technology, Shanghai, China). *Osmanthus* plants treated with a solution containing 1% ethanol served as the blank control. TIBA was sprayed every 24 h, and sample collection was performed every 24 h, with three biological replicates for each sample.

### Sequence analysis and *cis*-acting element analysis of promoters

We generated a phylogenetic tree and aligned multiple sequences using MEGA version 5.0 from Mega Ltd (Auckland, New Zealand) and DNAMAN version 5.2.2 from Lynnon Biosoft Bioinformatic Solutions (San Ramon, CA, USA). We also conducted an identity search for translated amino acids using the NCBI BLAST network server, available at https://blast.ncbi.nlm.nih.gov/Blast.cgi. Promoter sequences were obtained from the genomic DNA of *O. fragrans* ‘Liuyejingui’, sourced from the *O. fragrans* genome database. These sequences were then subjected to analysis for the prediction of *cis*-acting elements using the PlantCARE tool, accessible at http://bioinformatics.psb.ugent.be/webtools/plantcare/html/.

### Promoter analysis

Genomic DNA was extracted from leaves of *O. fragrans* ‘Yanhong’ using the Plant Genomic DNA Kit from Tiangen, China. Subsequently, DNA quality and concentration were determined using the 2100 Bioanalyzer RNA Nanochip from Agilent (Santa Clara, CA, USA). The 982-bp promoter region upstream of the ATG start codon of OfROS1a was amplified and then inserted into the pCAMBIA1300-GUS vector at the HindIII*/*BamHI sites, enabling regulation of GUS reporter gene expression. The resulting construct, termed *OfROS1a*pro::GUS, was transiently transformed into *N. benthamiana* leaves using the *Agrobacterium*-mediated method. To detect GUS activity, samples were collected from a minimum of five leaves, rapidly frozen in liquid nitrogen, and then pulverized in GUS extraction buffer. After centrifugation, the resulting clear supernatants were subjected to detection using a GloMax^®^ multifunctional instrument from Promega (Madison, WI, USA). Primer details can be found in Supplementary Data Table S13.

### Generation of transgenic petunia

The full-length cDNA of 2643 bp for *OfROS1a* was amplified via PCR. Subsequently, the entire coding sequence of *OfROS1a*, excluding the stop codon, was integrated into the pORE-R4-35AA vector using primers that contained NheI and XhoI restriction sites, resulting in the construction of the 35S::OfROS1a vector [88]. *Petunia hybrida* cv. ‘Mitchell Diploid’ (MD) was used as the genetic background to generate transgenic plants. The binary vectors were introduced into *Agrobacterium tumefaciens* strain EHA105 using electroporation. Subsequently, *P. hybrida* leaf discs were transformed with this bacterial strain, as reported previously [89]. The primers are listed in Supplementary Data Tables S14 and S15.

### Statistical analysis

Statistical analyses of the data were performed using a one-way ANOVA followed by a Duncan’s multiple range test and Student’s *t*-test with at least three replicates. *P* values ≤0.05 were considered to indicate significance.

## Supplementary Material

Web_Material_uhae010

## Data Availability

Reference genomes of *O. fragrans* are available as public genome data at the National Center of Biotechnology Information (NCBI) (https://www.ncbi.nlm.nih.gov/genome/?term=txid93977[orgn]) [44]. Whole-genome bisulfite sequencing and transcriptome sequencing data for floral buds subjected to diverse treatment durations at 19°C in comparison with those treated at 23°C have been deposited in the public NCBI BioProject database under accession numbers PRJNA1014796 and PRJNA1014987. Transcriptome sequencing data for corollas of *OfROS1a* overexpression and controls have been deposited in the public NCBI BioProject database under accession number PRJNA1014991. Reference genomes of *P. hybrida* were obtained from the public genome website (https://solgenomics.net/ftp/genomes/Petunia_axillaris/assembly/Petunia_axillaris_v1.6.2_genome.fasta).
