# Supplementary material for: DNA methylome analysis reveals novel insights into active hypomethylated regulatory mechanisms of temperature-dependent flower opening in Osmanthus fragrans
Source: Hortic Res. 2024 Jan 10;11(3):uhae010. doi: 10.1093/hr/uhae010 (PMC10923647; doi:10.1093/hr/uhae010)
Supplement: Web_Material_uhae010 [file web_material_uhae010.zip › Supplementary Figures revised.docx]

**Supplementary Figures**

**Title:** DNA methylome analysis reveals novel insights into active hypomethylated regulatory mechanisms of temperature-dependent flower opening in *Osmanthus fragrans*

Shiwei Zhong^1#^, Huijun Zhu^1#^, Wenle Li^1^, Dan Wu^1^, Yunfeng Miao^1^, Bin Dong^1^, Yiguang Wang^1^, Zheng Xiao^1^, Qiu Fang^1^, Jinping Deng^1^, Hongbo Zhao^1^
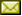


1. Zhejiang Provincial Key Laboratory of Germplasm Innovation and Utilization for Garden Plants, Key Laboratory of National Forestry and Grassland Administration on Germplasm Innovation and Utilization for Southern Garden Plants, School of Landscape and Architecture, Zhejiang A&F University, Hangzhou, Zhejiang 311300, China.

**Address**: School of Landscape Architecture, Zhejiang Agriculture and Forestry University, Hangzhou 311300, Zhejiang, China

**Name of correspondence author**: Hongbo Zhao

**Supplementary information**: 10 figures.


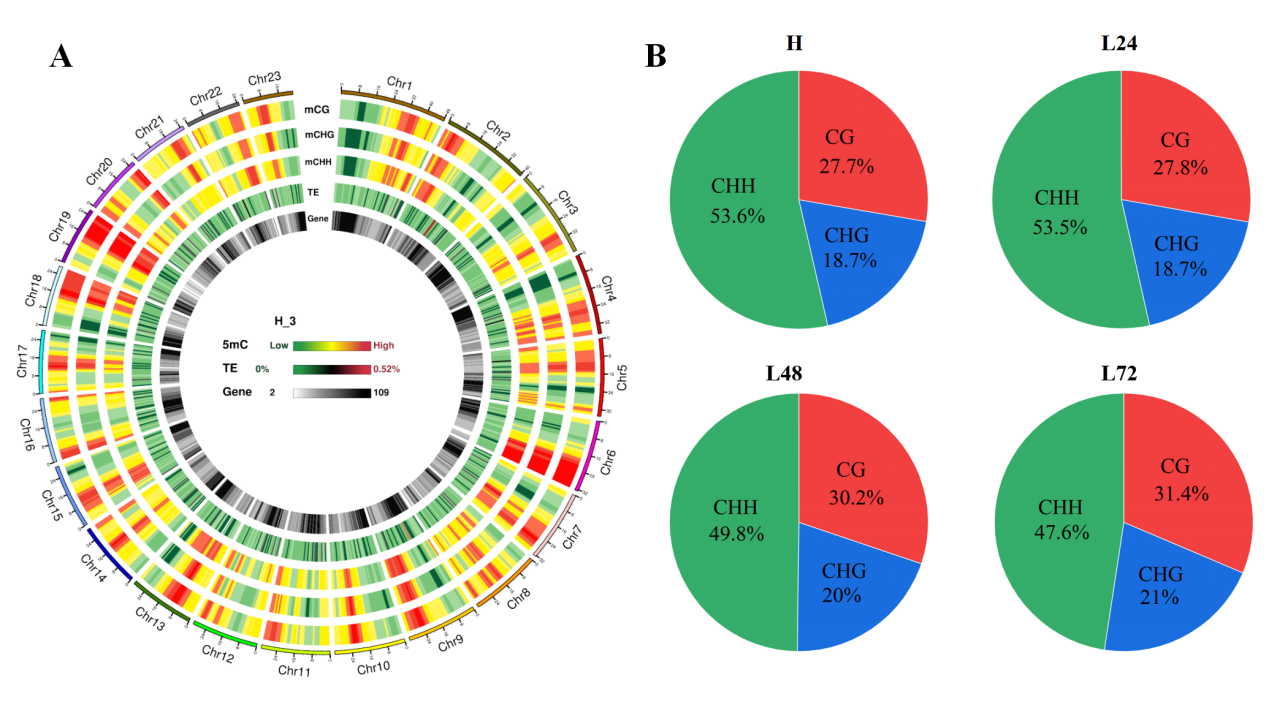


Figure S1. The epigenome of sweet osmanthus cv. ‘Yanhong Gui’. (A) Circos plots of DNA methylation density within 23 chromosomes of Osmanthus fragrans. Track order (from outside to inside): density plot of 5mC in CG, CHG, and CHH contexts; density of transposable elements (TEs); gene density of each chromosome. (B) Relative proportions of mCs in three sequence contexts (CG, CHG and CHH) in floral buds treated under 19°C for 24 (L24), 48 (L48), 72 (L72) hours. The floral buds treated under 23°C (H) were used as controls.


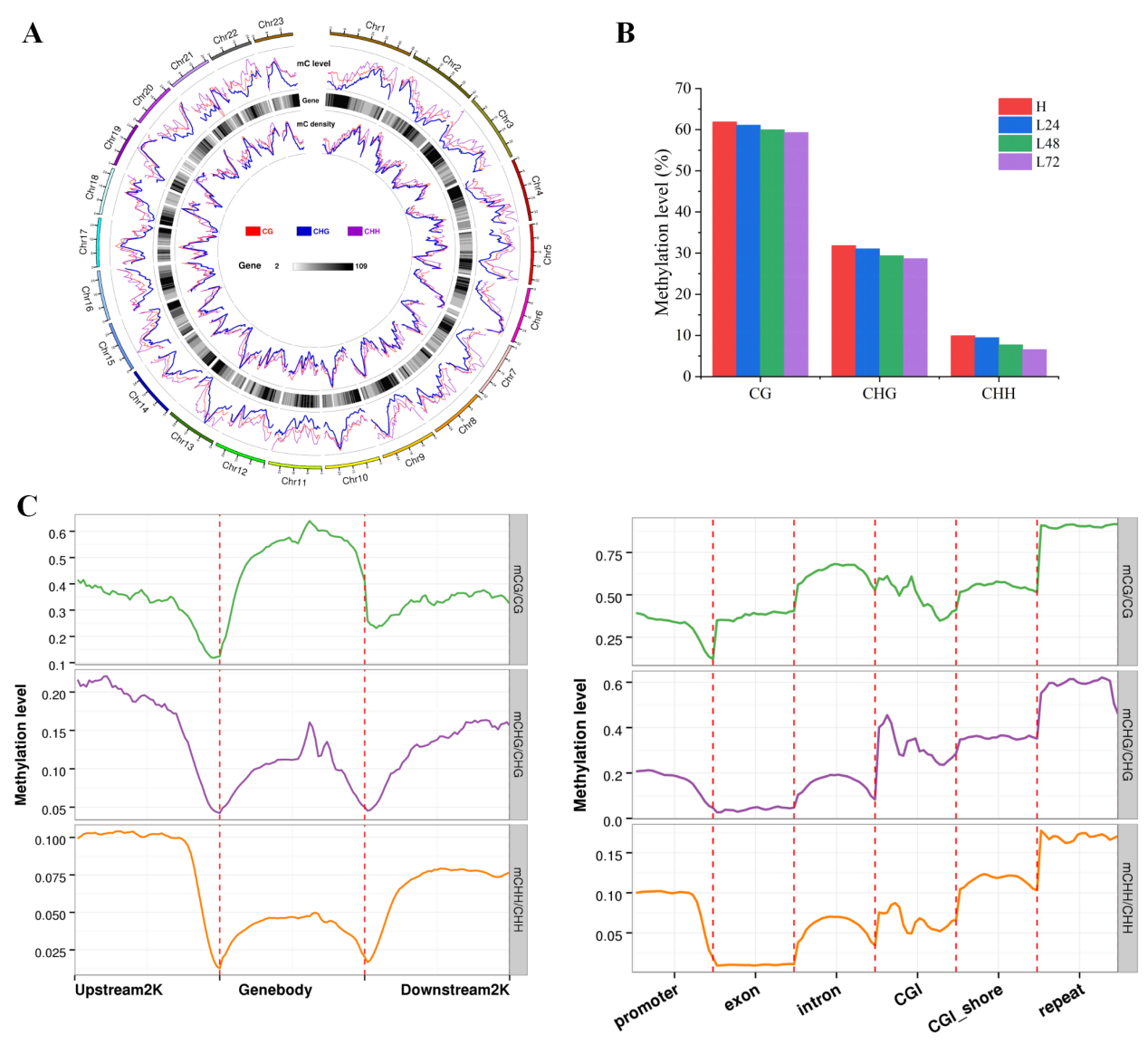


Figure S2. Methylation characterization of sweet osmanthus cv. ‘Yanhong’ thoughout the genome. (A) DNA methylation levels of 5mC in CG, CHG and CHH contexts within 23 chromosomes of Osmanthus fragrans. Track order (from outside to inside): methylation level; heat map of gene density; density of mC context. (B) Fractional methylation levels of the CG, CHG and CHH contexts in floral buds with different treatments. (C) DNA methylation levels of CG, CHG, and CHH for genes (left) and various genomic regions including promoter, exon, intron, CpG island named CGI, 2 kb upstream and downstream of CGI named CGI_shore, and repeat region (right).


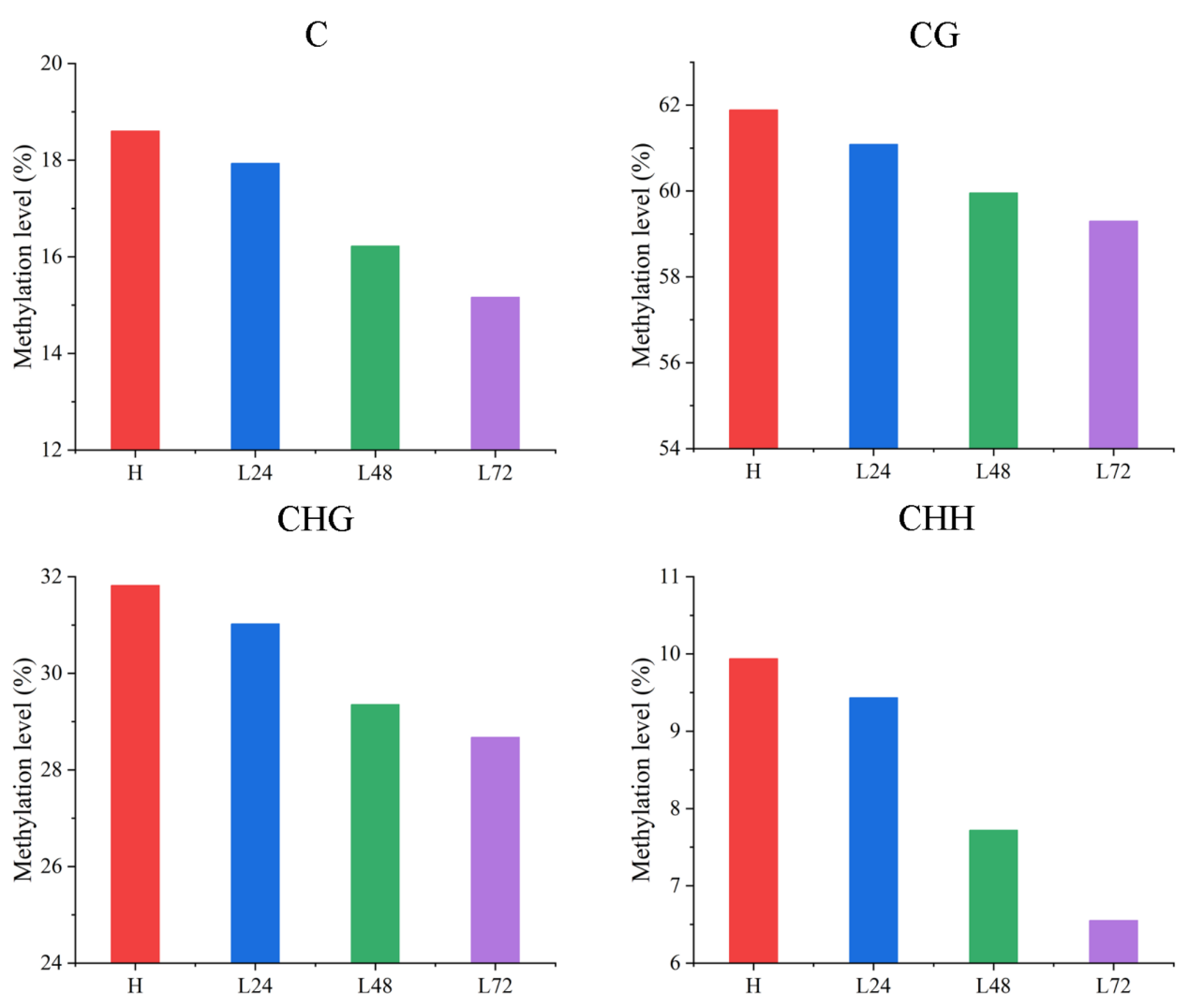


Figure S3. The mean methylation levels of mC, mCG, mCHG and mCHH in floral buds treated under 19°C for 24 (L24), 48 (L48), 72 (L72) hours, and in floral buds that failed to open treated under 23°C (H) used as controls in *Osmanthus fragrans*.


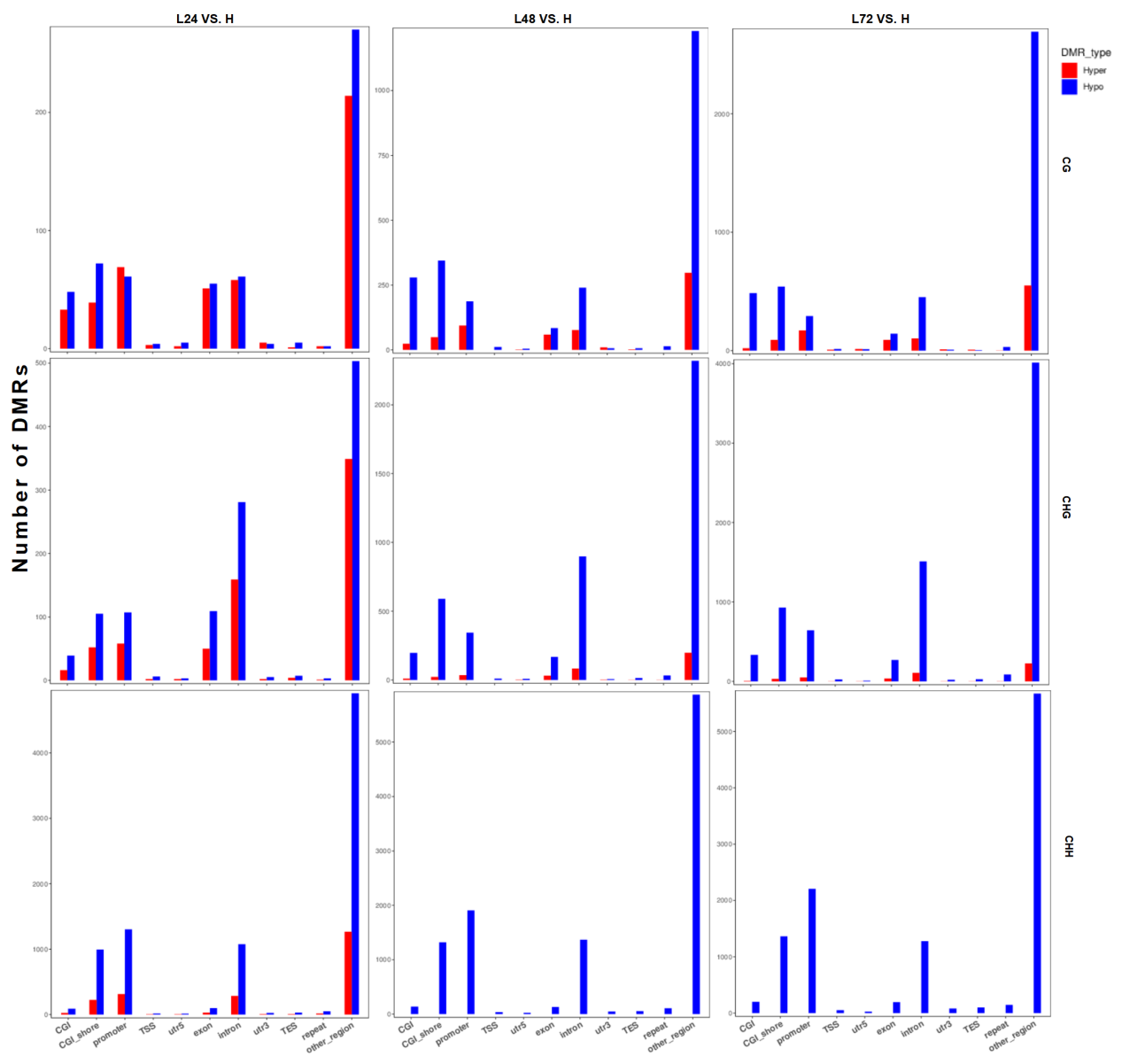


Figure S4. Number of DMRs, including hyper/hypomethylated sequences in the CGI, CGI shore, promoter, TSS, 5’UTR, exon, intron, 3’UTR, TES, repeat and other region, among different (L24 VS. H, L48 VS. H, L72 VS. H) comparisons in the CG, CHG and CHH contexts.


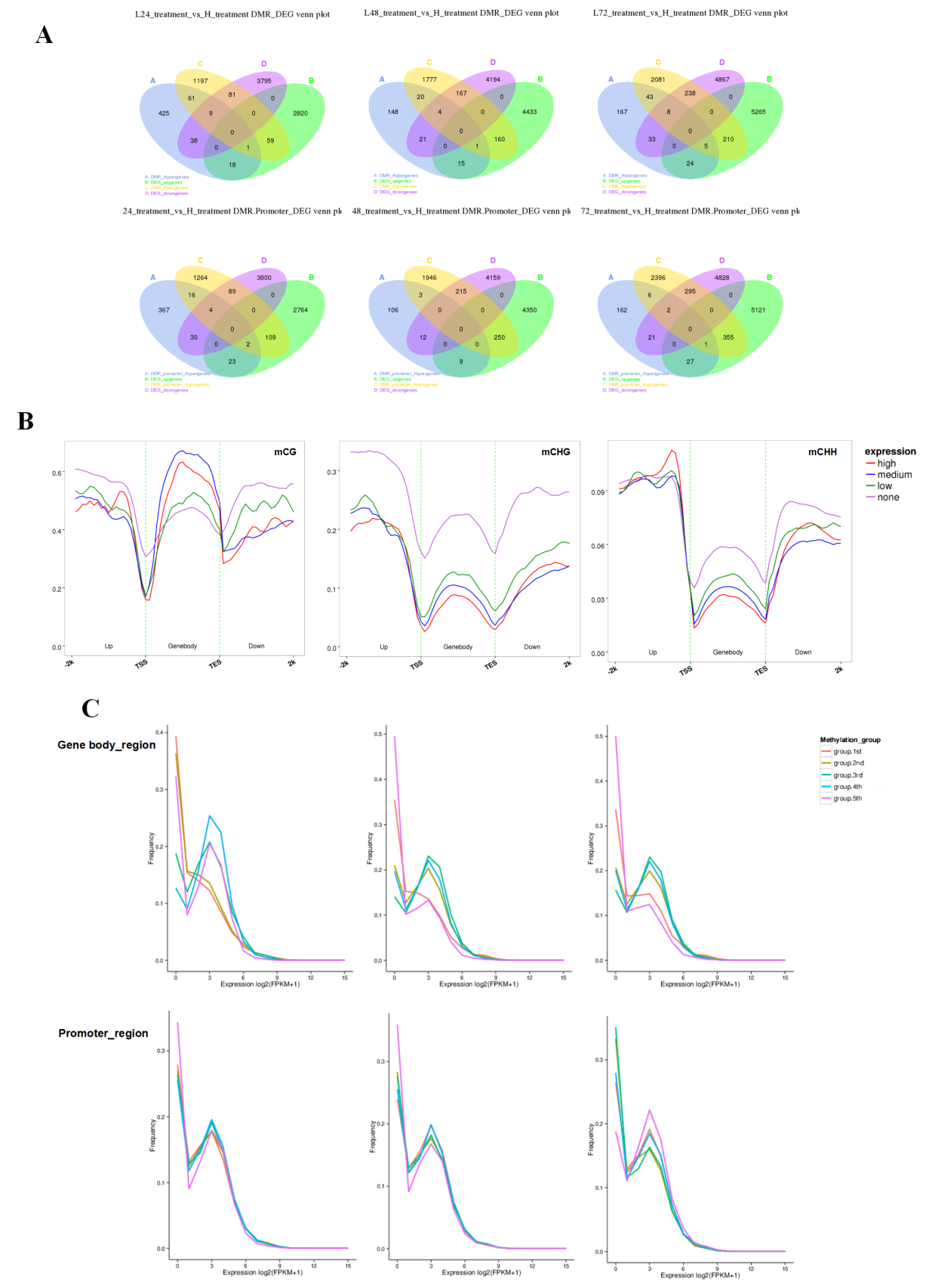


Figure S5. Correlation between DNA methylation and gene expression. (A) Venn diagrams indicating unique and common DEGs, DMR_genes, and DMR_promoter_genes in the three comparisons. (B) Distributions of methylation levels within gene bodies by different expression levels: none [genes with fragments per kilobase of transcript per million (FPKM) < 1 were considered unexpressed], low, medium, and high. (C) Expression profiles of different methylated levels. Methylated genes were divided into five groups based on promoter and gene body methylation levels: group.1st is the lowest and group.5th is the highest.


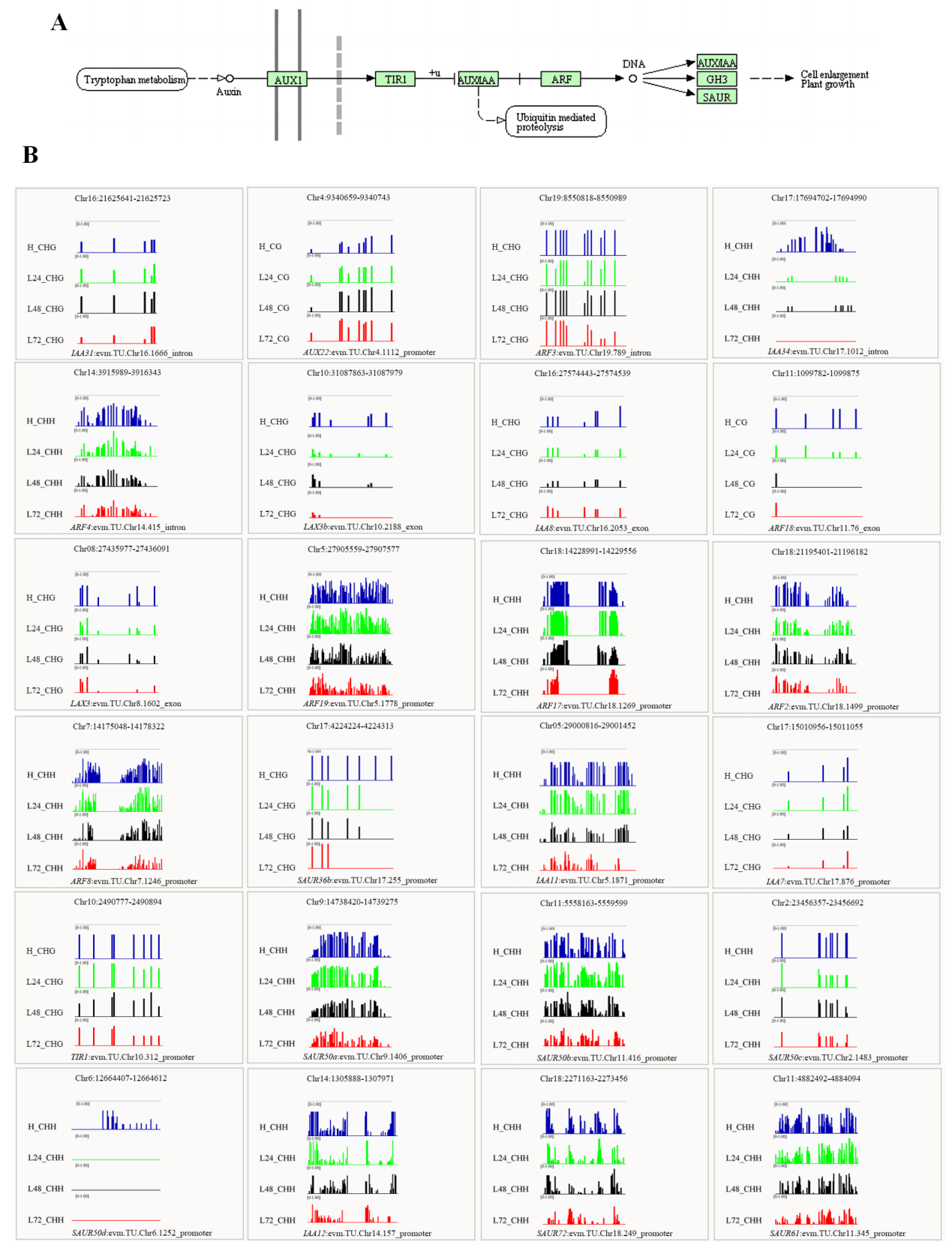


Figure. S6 IGV snapshots of the representative hypomethylated and hypermethylated DMRs related to EDGs of auxin signal transduction. (A) Pathway of auxin signal transduction. (B) IGV snapshots of the representative hypomethylated and hypermethylated DMRs with diverse treatment durations at 19°C.


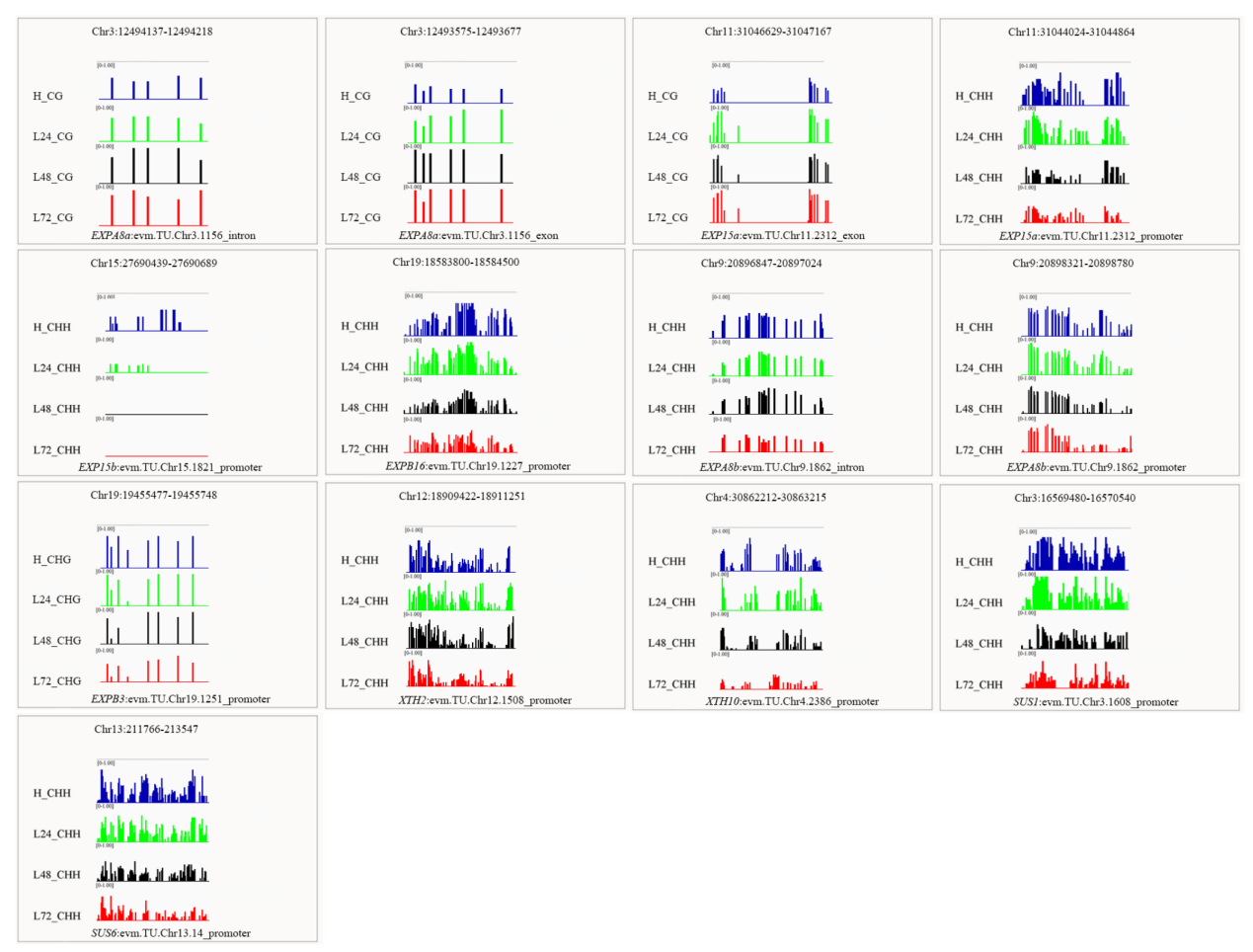


Figure. S7 IGV snapshots of the representative hypomethylated and hypermethylated DMRs related to EDGs of flowering with diverse treatment durations at 19°C.


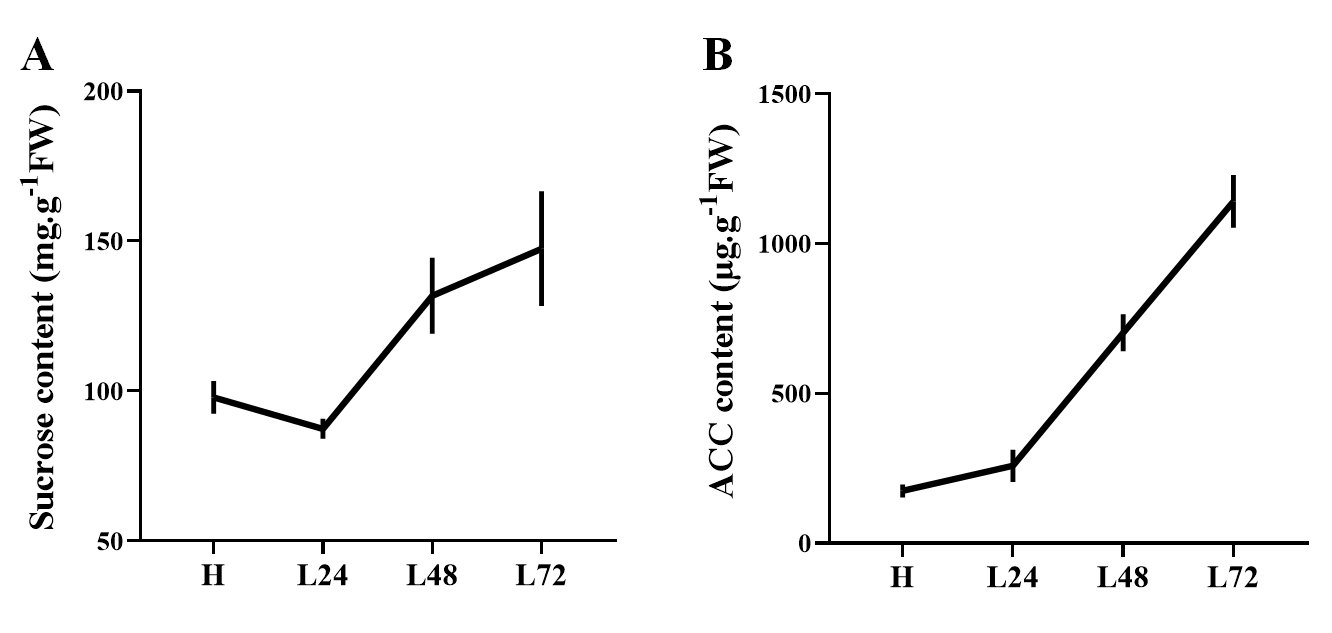


Figure. S8 Sucrose and 1-Aminocyclopropane-1-carboxylic acid (ACC) contents in floral buds with diverse treatment durations at 19°C in *Osmanthus fragrans*.


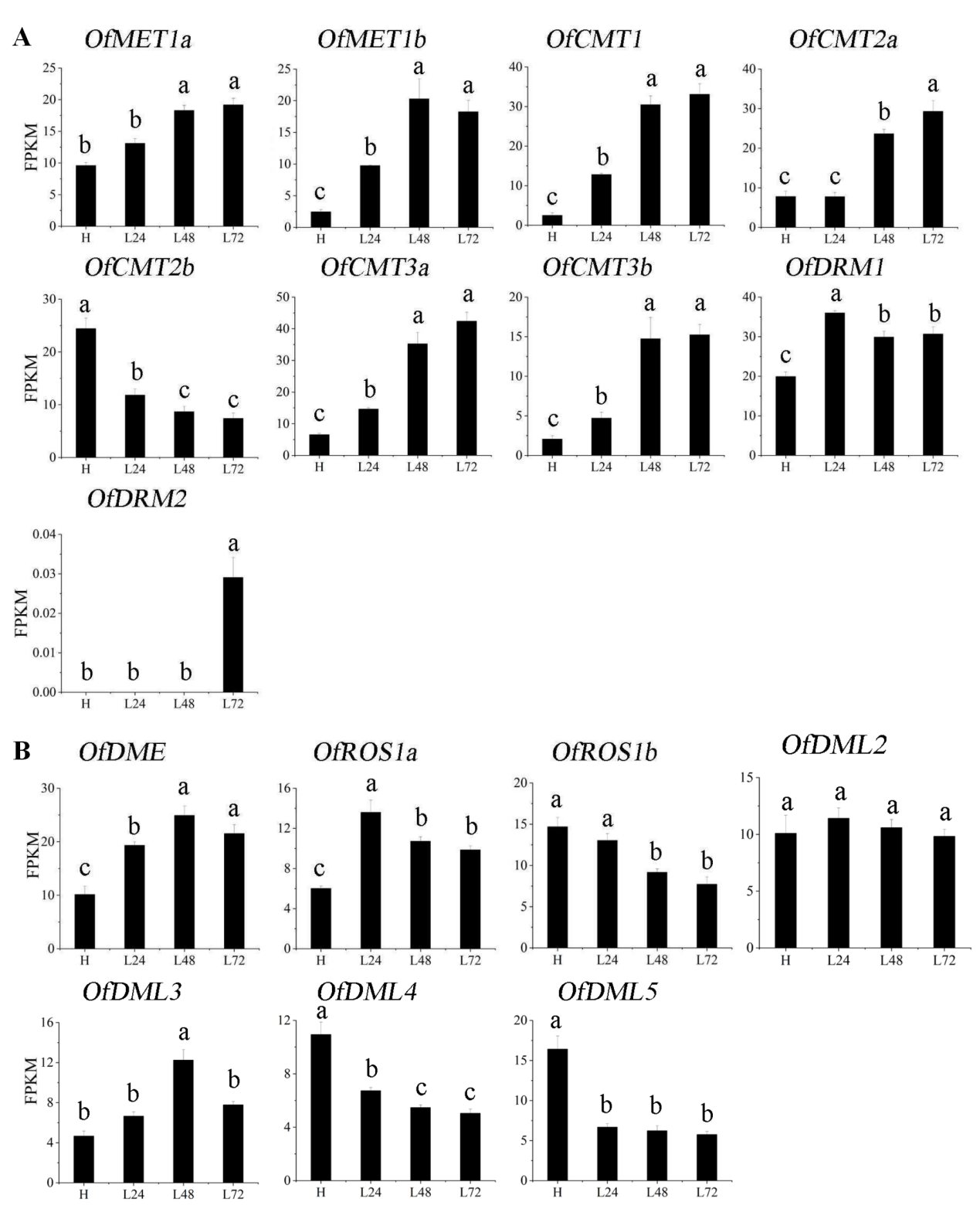


Figure S9. Expression of DNA methyltransferase genes and DNA demethylase genes of floral buds under high/low temperature-response period in Osmanthus fragrans. (A-B) Transcript levels of DNA methyltransferase genes (A) and DNA demethylase genes (B) in the floral buds treated at 19°C for 24 (L24), 48 (L48), 72 (L72) h and the floral buds under treatment of 23°C (H) used as controls. The data was presented as the means ± SEs (n=3). The statistical analysis was performed using one-way analysis of variance (ANOVA) followed by Duncan’s multiple range test (DMRT) with three biological replicates. P values ≤0.05 were considered signifcant.


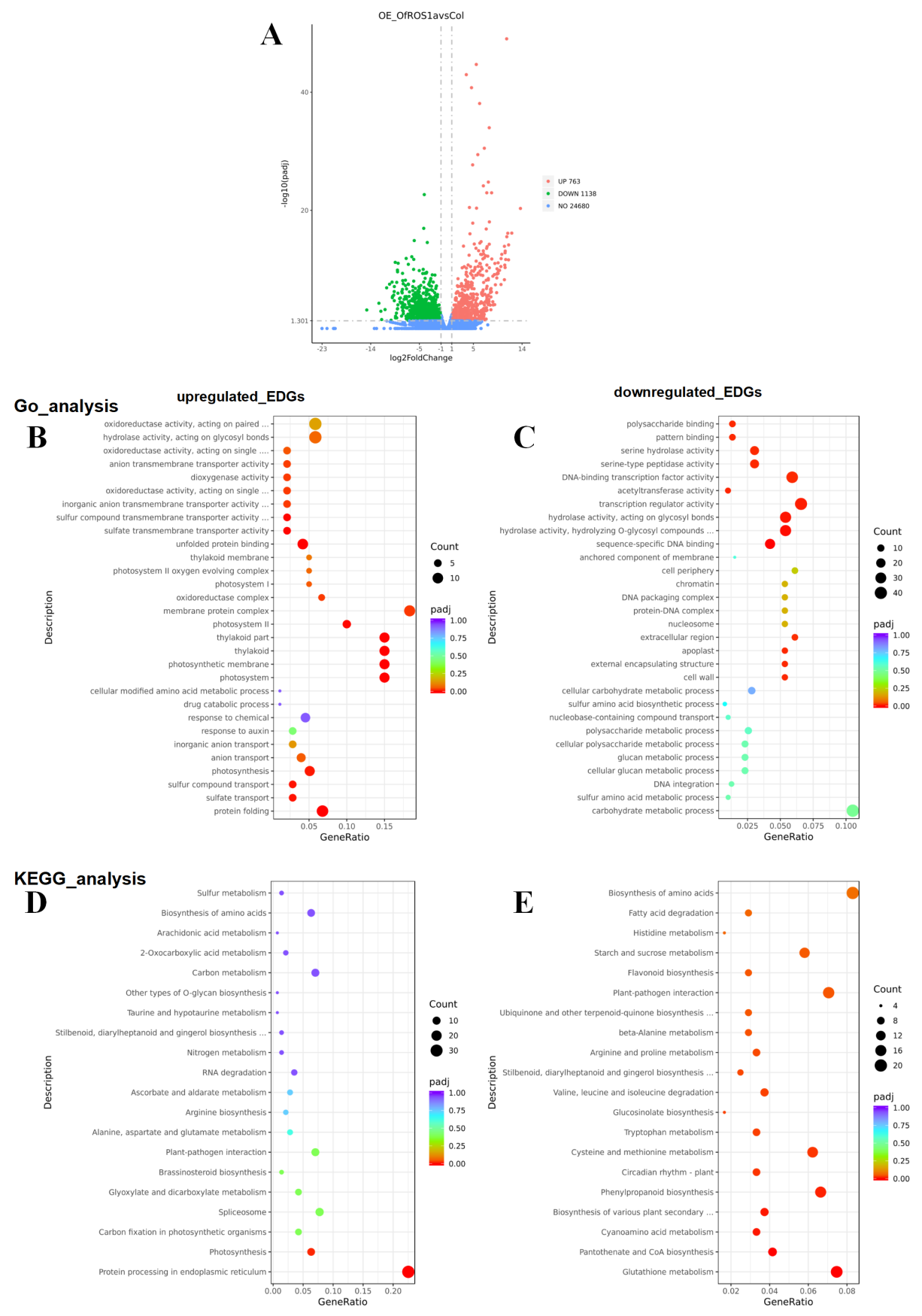


Figure S10. Analysis of the differential expressed genes in *OfROS1a*-overexpressed petunia corollas compared with controls. (A) Volcano plot, the green dots in the figure represent the genes with transcription that were decreased, the red dots represent those were increased, and the black dots indicates genes with no significant difference. (B-C) Gene ontology (GO) analysis indicated that the eneichment of members were significantly up-regulated (B), and were significantly down-regulated (C). (D-E) KEGG analysis indicated that the enrichment of members were significantly up-regulated (D), and were significantly down-regulated (E).
